# *APOE-***ε***4* synergizes with sleep disruption to accelerate A**β** deposition and A**β**-associated tau seeding and spreading

**DOI:** 10.1172/JCI169131

**Published:** 2023-07-17

**Authors:** Chanung Wang, Aishwarya Nambiar, Michael R. Strickland, Choonghee Lee, Samira Parhizkar, Alec C. Moore, Erik S. Musiek, Jason D. Ulrich, David M. Holtzman

**Affiliations:** Department of Neurology, Hope Center for Neurological Disorders, Knight Alzheimer’s Disease Research Center, Washington University School of Medicine, St. Louis, Missouri, USA.

**Keywords:** Neuroscience, Alzheimer disease, Lipoproteins, Neurodegeneration

## Abstract

Alzheimer’s disease (AD) is the most common cause of dementia. The *APOE*-ε4 allele of the apolipoprotein E (*APOE*) gene is the strongest genetic risk factor for late-onset AD. The *APOE* genotype modulates the effect of sleep disruption on AD risk, suggesting a possible link between apoE and sleep in AD pathogenesis, which is relatively unexplored. We hypothesized that apoE modifies Aβ deposition and Aβ plaque–associated tau seeding and spreading in the form of neuritic plaque–tau (NP-tau) pathology in response to chronic sleep deprivation (SD) in an apoE isoform–dependent fashion. To test this hypothesis, we used APPPS1 mice expressing human *APOE*-ε3 or -ε4 with or without AD-tau injection. We found that SD in APPPS1 mice significantly increased Aβ deposition and peri-plaque NP-tau pathology in the presence of APOE4 but not APOE3. SD in APPPS1 mice significantly decreased microglial clustering around plaques and aquaporin-4 (*AQP4*) polarization around blood vessels in the presence of APOE4 but not APOE3. We also found that sleep-deprived APPPS1:E4 mice injected with AD-tau had significantly altered sleep behaviors compared with APPPS1:E3 mice. These findings suggest that the *APOE*-ε4 genotype is a critical modifier in the development of AD pathology in response to SD.

## Graphical abstract

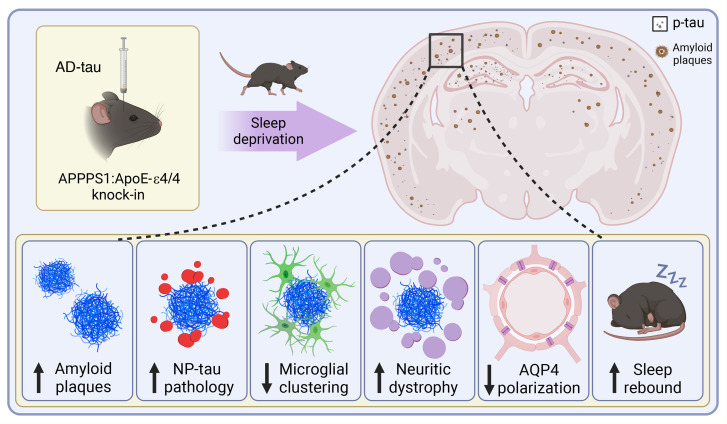

## Introduction

Alzheimer’s disease (AD) is the most common cause of dementia in the elderly and a major, growing public health problem. Currently, approximately 47 million people worldwide are living with dementia (1). Thus, identifying modifiable risk factors for AD is critical. AD is pathologically characterized by extracellular amyloid-β (Aβ) plaques and intraneuronal, aggregated, hyperphosphorylated tau that is present in neurofibrillary tangles, dystrophic neurites, and neuropil threads (2). In addition to tau and Aβ, a large percentage of patients with AD exhibit sleep disturbances (3, 4). Sleep disturbances are prevalent in patients with symptomatic AD and are a major risk factor for early institutionalization (5). Recent studies show that sleep disturbances are detectable years before the onset of cognitive impairment, during the preclinical phase of AD (6). In mouse models of Aβ-amyloidosis, sleep deprivation (SD) can accelerate Aβ deposition, while promoting sleep with an orexin receptor antagonist can reduce amyloid plaque pathology (7, 8). Previous work in our laboratory showed that secreted forms of Aβ and tau fluctuate in the brain interstitial fluid (ISF) and cerebrospinal fluid (CSF) with the sleep-wake cycle and that SD increases Aβ and tau levels in mouse ISF and in human CSF (7, 9). Furthermore, we showed that acute SD induces a stronger increase in extracellular tau than Aβ, and that chronic SD increases the spreading of tau aggregates (9). However, it is not clear how these sleep disturbances impact the progression of AD in the presence of other risk factors such as different apolipoprotein (*APOE*) genotypes.

ApoE is a major lipid and cholesterol transporter in the brain. There are 3 common APOE alleles in humans: *APOE*ε*2*, *APOE*ε*3*, and *APOE*ε*4* (10). The *APOE*-ε4 genotype is strongly associated with increased AD risk and amyloid plaque accumulation in humans as compared with *APOE*ε3, whereas *APOE*ε*2* reduces the risk of AD (11). Prior studies have shown that *APOE*-ε4 has deleterious effects on amyloid aggregation, plaque deposition, and tau-mediated neurodegeneration (12–14). There are several human studies that found links between sleep and the *ApoE4* genotype. A study reported that the *APOE4* genotype is strongly associated with sleep-disordered breathing (15). Yaffe and colleagues found in older cognitively normal women, those with sleep-disordered breathing compared with those without sleep-disordered breathing had an increased risk of developing cognitive impairment (16). Another study reported that individuals with the *APOE4* genotype have an increased risk of developing obstructive sleep apnea (OSA) (17). OSA causes both intermittent hypoxia and sleep disturbance and is estimated to affect 1%–4% of middle-aged adults (18) and 24%–30% of elderly individuals (19). Another recent study showed that disrupted sleep was more frequent in males than in females and in carriers of the *APOE4* genotype than in noncarriers (20). They also reported that the influence of the *APOE4* genotype on sleep disturbances depends on the severity of the AD load. These studies suggest that, in humans, APOE4 is associated with disrupted sleep that could be linked to AD pathogenesis (21).

In this study, we investigated the interactions between the *APOE* genotype, SD, Aβ, and Aβ-induced tau seeding and spreading using APPPS1 mice that express APOE3 or APOE4. We hypothesized that chronic SD would accelerate Aβ plaque deposition in an APOE isoform–dependent manner and would also increase seeded neuritic plaque tau (NP-tau) pathology. Specifically, we hypothesized that the *APOE*-ε4 genotype exacerbates the effects of SD and Aβ pathology and Aβ-induced tau seeding and spreading in the form of NP-tau pathology. We also hypothesized that Aβ and tau pathology in the presence of ApoE4 would be associated with altered sleep-wake behavior.

## Results

### SD exacerbates Aβ plaque deposition in the presence of APOE4 but not APOE3.

**Figure 1:**
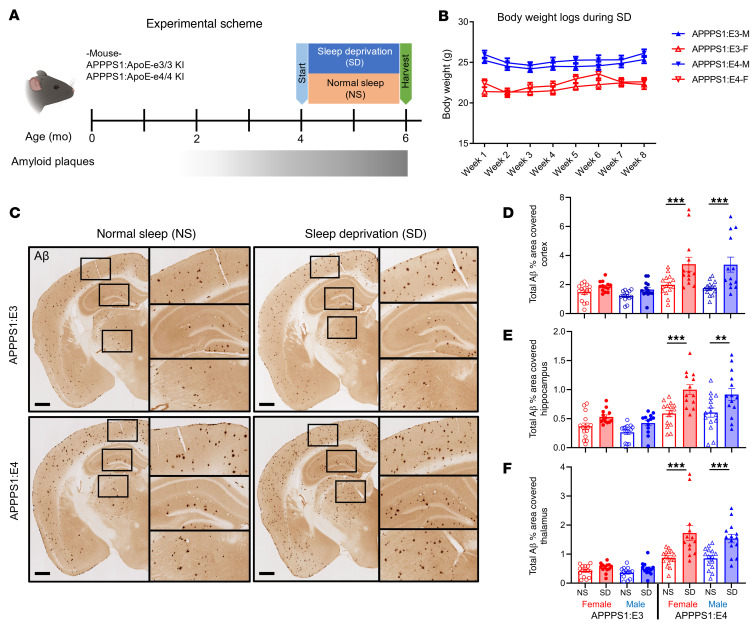
SD exacerbates amyloid plaque deposition in the presence of apoE4 but not apoE3. (**A**) Schematic of the experimental design. Four-month-old APPPS1:E3 and APPPS1:E4 mice were placed in an automated sleep fragmentation chamber or a normal cage (*n* = 12–15 per group). (**B**) Body weight logs under the SD condition. (**C**) Representative images of anti–Aβ antibody–stained (HJ3.4-biotin) brain sections from APPPS1:E3 and APPPS1:E4 mice from the NS and SD groups. Scale bars: 500 μm. Original magnification, ×1.25 (insets). (**D**–**F**) Quantification of the percentage of area covered by Aβ staining in cortex (**D**), hippocampus (**E**), and thalamus (**F**). Data are presented as the mean ± SEM. Significance was determined by 3-way ANOVA with Šidák’s multiple-comparison test (sex, apoE genotype, and sleep condition). There was a significant effect of the apoE genotype and sleep condition but not of sex. **P < 0.01 and ***P < 0.001. No other statistical comparisons were significant unless indicated. See also Supplemental Table 1.

**Figure 2:**
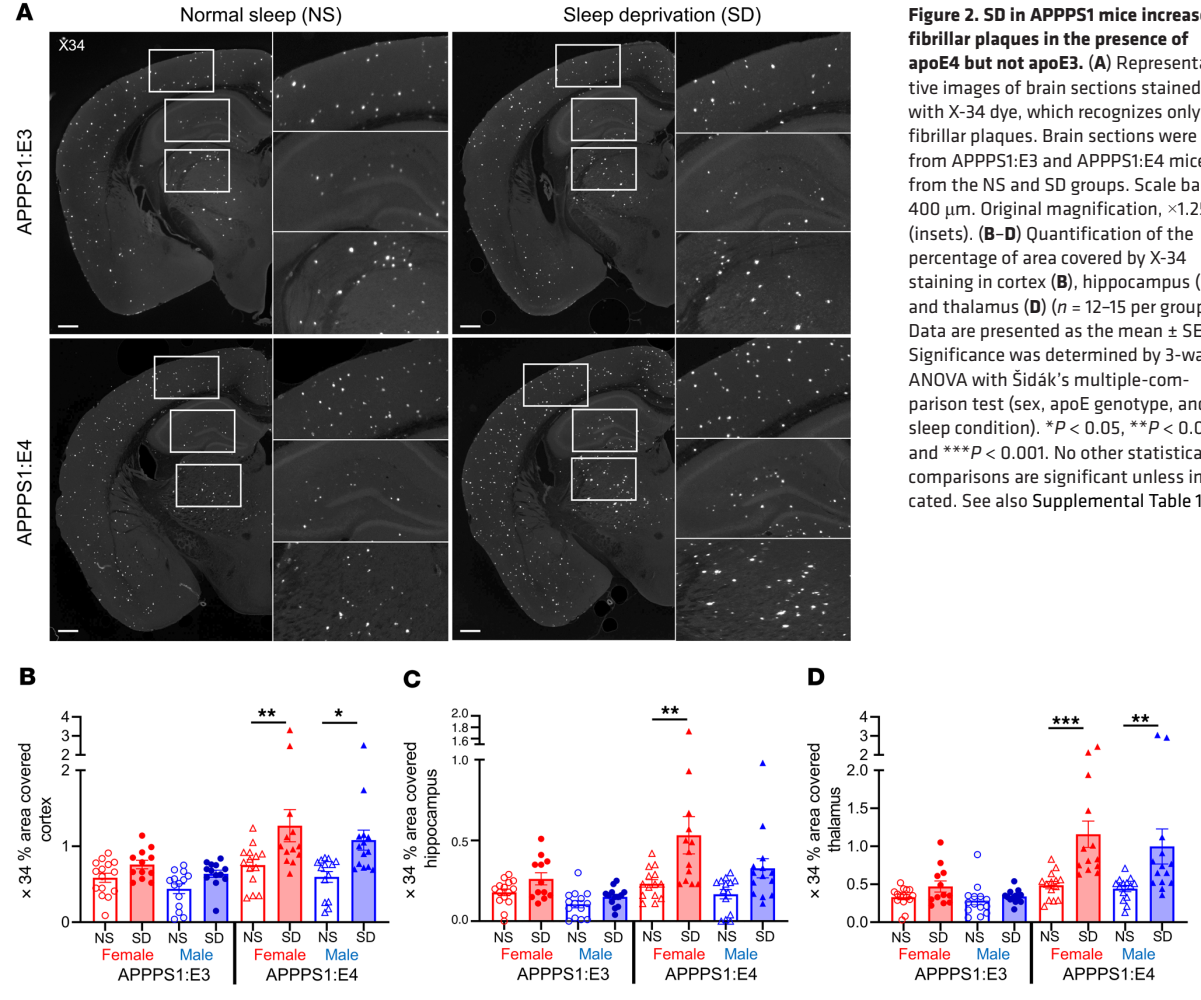
SD in APPPS1 mice increases fibrillar plaques in the presence of apoE4 but not apoE3. (**A**) Representative images of brain sections stained with X-34 dye, which recognizes only fibrillar plaques. Brain sections were from APPPS1:E3 and APPPS1:E4 mice from the NS and SD groups. Scale bars: 400 μm. Original magnification, ×1.25 (insets). (**B**–**D**) Quantification of the percentage of area covered by X-34 staining in cortex (**B**), hippocampus (**C**), and thalamus (**D**) (*n* = 12–15 per group). Data are presented as the mean ± SEM. Significance was determined by 3-way ANOVA with Šidák’s multiple-comparison test (sex, apoE genotype, and sleep condition). *P < 0.05, **P < 0.01, and ***P < 0.001. No other statistical comparisons are significant unless indicated. See also Supplemental Table 1.

Previously, we found that chronic SD significantly increased Aβ plaque deposition in 2 mouse models of β-amyloidosis that expressed murine Apoe (7). Murine and human apoE proteins are approximately 70% identical at the protein level and have functional differences (22). To determine whether human APOE isoforms influence SD-induced exacerbation of Aβ pathology, we studied APPPS1 mice expressing human APOE3 or APOE4 (APPPS1:E3 and APPPS1:E4) (23). Mice were sleep deprived for 8 weeks, beginning at 4 months of age (Figure 1A), a time point when these mice have a moderate amount of Aβ pathology in the cortex. To test the effects of chronic SD on overall health, we measured the body weight of sleep-deprived mice weekly and found no changes in body weight (Figure 1B). Chronic SD significantly increased Aβ plaque deposition in the cortex, hippocampus, and thalamus of APPPS1:E4, but not APPPS1:E3, mice (Figure 1, C–F). In each brain region, the overall Aβ plaque burden increased by approximately 1.8-fold in SD male and female APPPS1:E4 mice compared with non-sleep-deprived (normal sleep [NS]) control mice (Figure 1, D–F). Similarly, we found that APPPS1:E4, but not in APPPS1:E3, mice with chronic SD had significantly increased X34^+^ fibrillar Aβ plaques in (Figure 2). These findings suggest that APOE4, but not APOE3, synergizes with chronic SD to exacerbate Aβ plaque deposition.

### SD differentially affects microglial, and to some extent, astrocyte clustering and dystrophic neurite formation around plaques in an apoE isoform–dependent manner.

**Figure 3:**
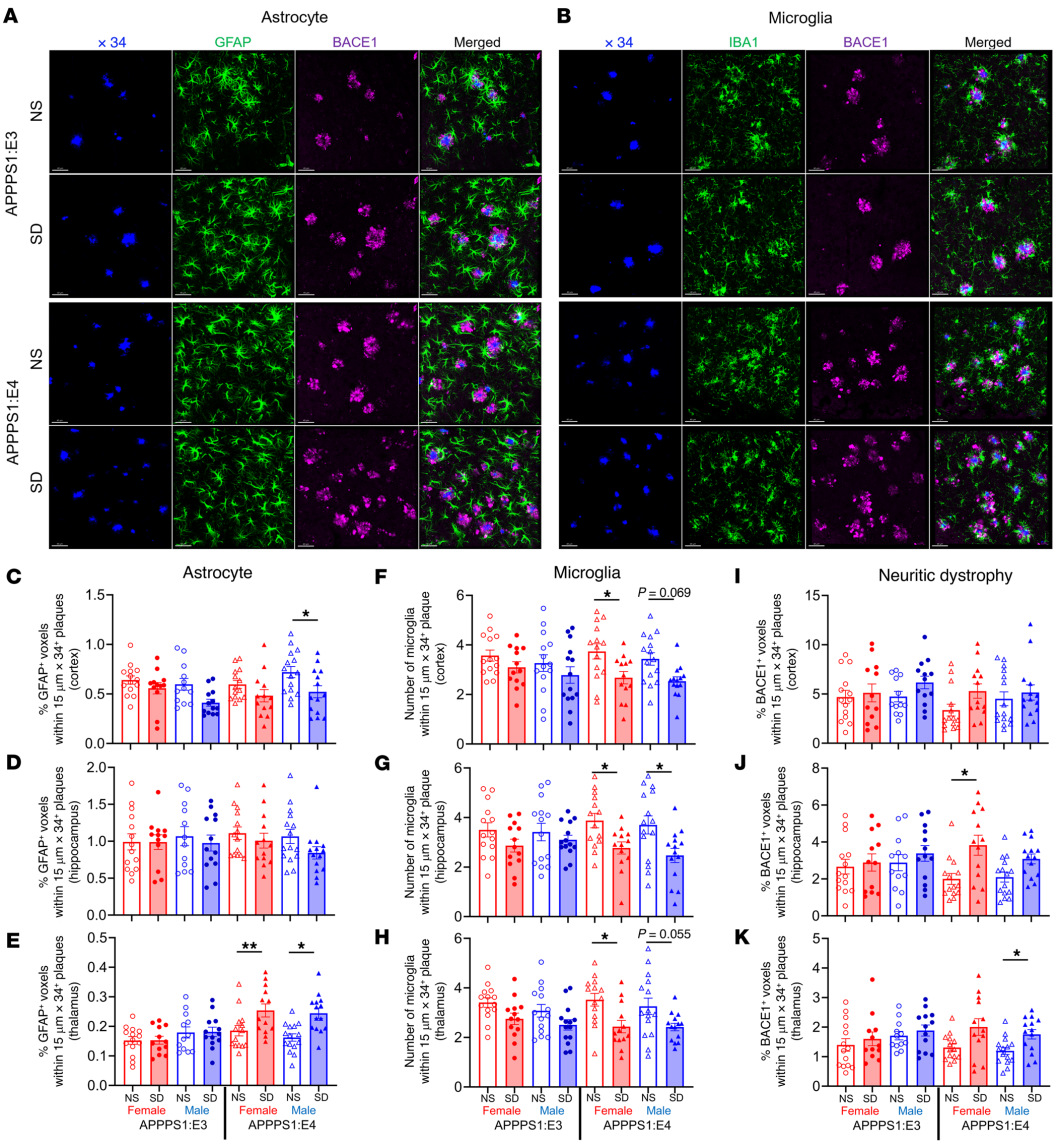
SD in APPPS1 mice differently affects the astrocyte population, microglia clustering, and dystrophic neurite formation around plaques in an apoE isoform–dependent manner. (**A**) Confocal images of GFAP-labeled astrocytes (green) and neuritic dystrophy (BACE1, magenta costained around X34^+^ plaques (blue) in cortex. Scale bars: 40 μm. (**B**) Confocal images of IBA1-labeled microglia (green) and neuritic dystrophy (BACE1, magenta) costained around X34^+^ plaques (blue) in hippocampus. Scale bars: 40 μm. (**C**–**E**) Quantification of the percentage of GFAP^+^ voxels within 15 μm of plaques in cortex (**C**), hippocampus (**D**), and thalamus (**E**) from APPPS1:E3 and APPPS1:E4 mice from the NS and SD groups (*n* = 12–15 per group). (**F**–**H**) Quantification of the number of microglial cells surrounding plaques in cortex (**F**), hippocampus (**G**), and thalamus (**H**) from APPPS1:E3 and APPPS1:E4 mice subjected to the NS or SD condition. (**I**–**K**) Quantification of the percentage of BACE1^+^ voxels within 15 μm plaques in cortex (**I**), hippocampus (**J**), and thalamus (**K**) from APPPS1:E3 and APPS1:E4 mice treated with the NS or SD condition. Data are presented as the mean ± SEM. Significance was determined by 3-way ANOVA with Sidak’s multiple-comparison test (sex, apoE genotype, and sleep condition). *P < 0.05 and **P < 0.01. See also Supplemental Table 1.

Previous research showed that 5 days of sleep restriction promotes astrocytic phagocytosis and microglial activation in mouse cerebral cortex to potentially remove overstimulated synapses (24). We therefore investigated how chronic SD affects astrocyte and microglia clustering around plaques in APPPS1 mice with different *APOE* genotypes (Figure 3, A and B). First, we observed that SD differently affected astrocyte clustering in brain regions in an apoE isoform–dependent manner (Figure 3, C–E). SD resulted in a decrease in glial fibrillary acidic protein (GFAP) astrocyte clustering around plaques in the cortex in sleep-deprived APPPS1:E4 male mice but not in female APPPS1:E4 mice as compared with the NS mice (Figure 3, C and D). In addition, GFAP^+^ astrocyte clustering around plaques was significantly increased in the thalamus of SD APPPS1:E4 mice but not in APPPS1:E3 mice (Figure 3E). Second, we found that clustering of ionized calcium–binding adaptor molecule 1 (IBA1) microglia around plaques was significantly decreased in SD APPPS1:E4 mice but not in APPPS1:E3 mice. This suggests that chronic SD in APPPS1 in the presence of APOE4 reduced microglial responsiveness to amyloid plaques (Figure 3, F–H, and Supplemental Figure 1, A and B; supplemental material available online with this article; https://doi.org/10.1172/JCI169131DS1). Given these results, we tested whether chronic SD differentially affects the formation of BACE1^+^ dystrophic neurites around plaques in an apoE isoform–dependent manner, since dystrophic neurite formation is strongly influenced by reactive microglia and their ability to cluster around plaques (25). Dystrophic neurites are abnormal neuronal processes characterized by aberrant swelling that accumulates various cellular organelles and cytoskeletal/signaling proteins that are present around amyloid plaques (25). We quantified BACE1^+^ dystrophic neurites around plaques and found that chronic SD induced an increase in neuritic dystrophy in the hippocampus of female APPPS1:E4 mice and in the thalamus in male APPPS1:E4 mice. We observed no significant changes in dystrophic neurites due to SD in APPPS1:E3 mice (Figure 3, I–K). These results suggest that in the presence of amyloid deposition, SD combined with apoE4 results in a dysfunctional microglial state resulting in less plaque-associated microglial clustering and phagocytosis and subsequently greater damage to surrounding neurites.

### SD increases Aβ-associated tau seeding and spreading in the presence of APOE4 but not APOE3.

**Figure 4:**
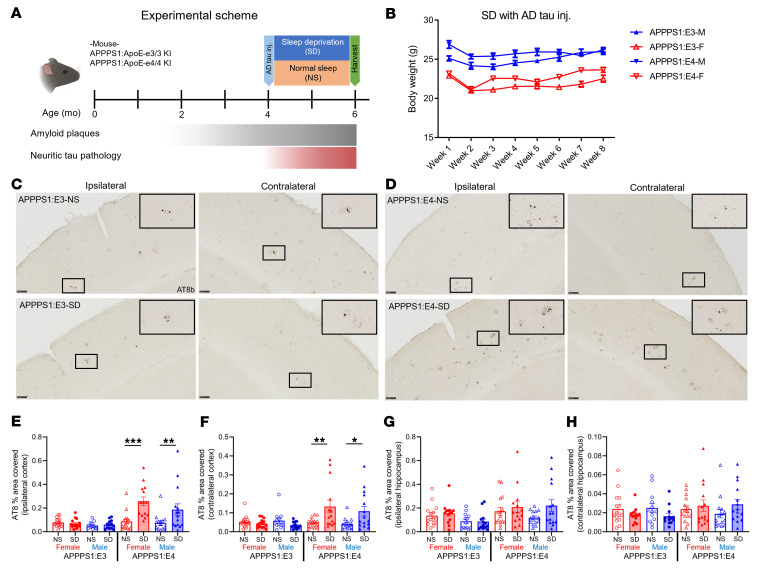
SD in APPPS1 mice significantly increases NP-tau seeding and spreading in cortex in the presence of APOE4 but not APOE3. (**A**) Schematic of the experimental design. Four-month-old APPPS1:E3 and APPPS1:E4 mice were injected with AD-tau in the hippocampus and overlaying cortex and were placed in sleep fragmentation chambers or normal cages for 8 weeks. All experimental mice were sacrificed at 6 months of age to evaluate tau seeding and spreading (*n* = 13–15 per group). (**B**) Body weight logs with AD-tau injection (inj.) during the SD condition. (**C** and **D**) Representative images of the ipsi- and contralateral hemisphere stained with AT8^+^ to identify NP-tau pathology in AD-tau–injected APPPS1:E3 (**C**) and APPPS1:E4 mice (**D**). Scale bars: 100 μm. Original magnification, ×10 (insets). (**E**–**H**) Quantification of the percentage of area covered by AT8^+^ staining in the ipsi- and contralateral cortices (**E** and **F**, respectively) and hippocampi (**G** and **H**, respectively) of AD-tau–injected APPPS1:E3 and APPPS1:E4 mice. Data are presented as the mean ± SEM. Significance was determined by 3-way ANOVA with Šidák’s multiple-comparison test (sex, apoE genotype, and sleep condition). *P < 0.05, **P < 0.01, and ***P < 0.001. See also Supplemental Table 1.

To determine whether APOE4 synergizes with SD to exacerbate Aβ-induced NP-tau pathology, we used a previously described model of amyloid-induced tau seeding and spreading by injecting mouse brains with sarkosyl-insoluble tau aggregates isolated from human AD brain tissue (AD-tau) (25–27). Four-month-old APPPS1:E3 and APPPS1:E4 mice were injected unilaterally with AD-tau into the dentate gyrus of the hippocampus and overlying cortex. After 3 days of recovery from the AD-tau injection and adaptation to the new environment, the mice were subjected to 8 weeks of SD or NS conditions (Figure 4A). We measured the body weight of SD mice weekly and found no changes in the body weight of AD-tau–injected mice with or without SD, indicating no major change in health or stress (Figure 4B). Using AT8 staining, which recognizes tau phosphorylation at both serine 202 and threonine 205 (Figure 4, C and D), we observed that chronic SD increased NP-tau seeding (ipsilateral) and spreading (contralateral) in the cortex of APPPS1:E4, but not APPPS1:E3, mice compared with the same mice under NS conditions (Figure 4, E and F). We also observed a nonsignificant trend toward increased NP-tau seeding and spreading in the hippocampus of sleep-deprived APPPS1:E4, but not APPPS1:E3, mice (Figure 4, G and H). SD also significantly increased Aβ plaque deposition and fibrillar Aβ plaques in an apoE isoform–dependent fashion in the cortex and thalamus following AD-tau injection (Supplemental Figures 2 and 3).

**Figure 5:**
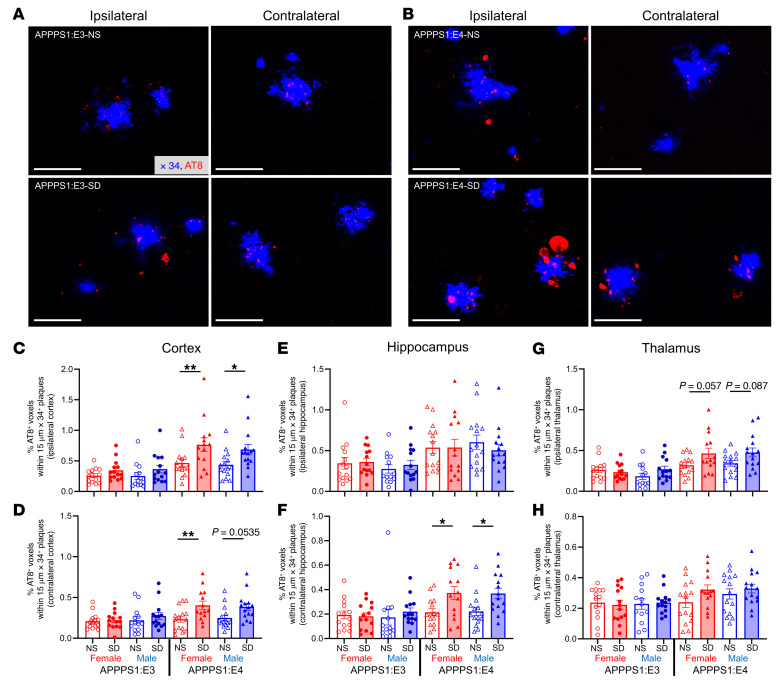
NP tau pathology is significantly increased with SD in the presence of apoE4 but not apoE3. (**A** and **B**) Confocal images of cortical AT8^+^ NP-tau (red) around X34^+^ plaques (blue) in the ipsi- and contralateral cortices of AD-tau–injected APPPS1:E3 (**A**) and APPPS1:E4 (**B**) mice (*n* = 13–15 per group). Scale bars: 40 μm. (**C**–**H**) Quantification of the percentage AT8^+^ volume within 15 μm plaques in the ipsi- and contralateral cortices (**C** and **D**, respectively), hippocampi (**E** and **F**, respectively), and thalami (**G** and **H**, respectively) from AD-tau–injected APPPS1:E3 and APPPS1:E4 mice. Data are presented as the mean ± the SEM. Significance was determined by 3-way ANOVA with Šidák’s multiple-comparison test (sex, apoE genotype, and sleep condition). *P < 0.05 and **P < 0.01. See also Supplemental Table 1.

Given that SD can increase overall plaque burden in the presence of E4 but not E3, which could in turn affect amyloid-associated tau seeding, we quantified the amount of NP-tau surrounding individual X34^+^ Aβ plaques (Figure 5, A and B). We confirmed that NP-tau pathology was significantly increased on a per-plaque basis in the ipsilateral cortex and thalamus but not in the hippocampus of APPPS1:E4 mice (Figure 5, C, E, and G). In contrast, no significant differences were detected in NP-tau pathology on a per-plaque basis in APPPS1:E3 mice. We also observed significantly increased NP-tau spreading at a per-plaque level in the contralateral cortex and hippocampus but not in the thalamus (Figure 5, D, F, and H). Interestingly, we observed that chronic SD did not alter NP-tau seeding on a per-plaque basis in the hippocampus of APPPS1:E4 mice, but there was significantly increased NP-tau spreading on a per-plaque basis (Figure 5F). These results are similar to those from our prior study, which showed that chronic SD in mice does not alter tau seeding in the hippocampus in the presence of murine apoE; however, it significantly increases tau spreading in a model of primary tauopathy (9). Overall, our findings suggest that chronic SD increases Aβ-associated tau seeding and spreading in an apoE isoform–dependent manner.

### SD significantly affects microglia clustering and neuritic dystrophy formation around plaques in an apoE-dependent manner.

**Figure 6:**
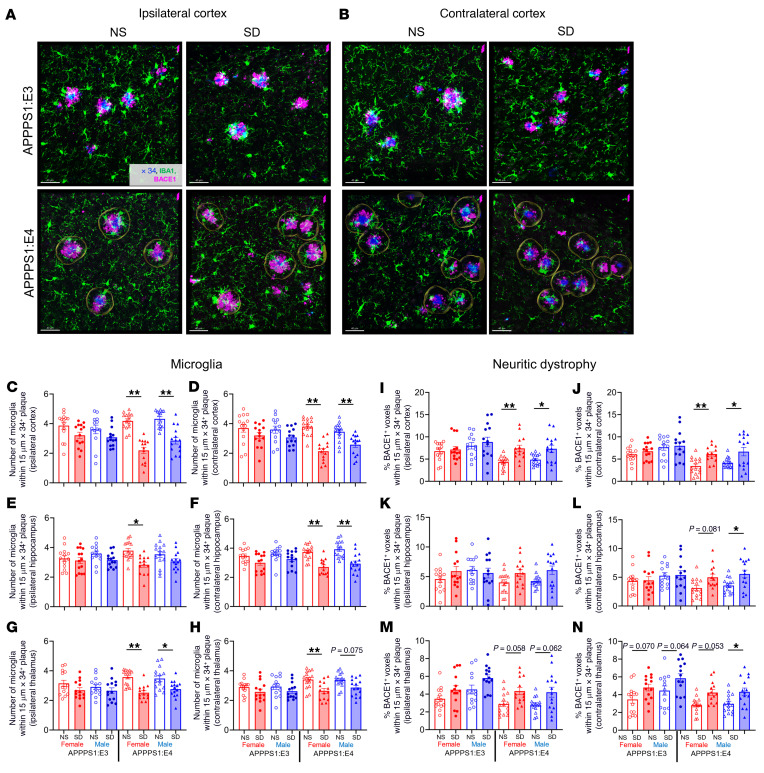
SD in AD-tau–injected APPPS1 mice significantly affects microglia clustering and neuritic dystrophy formation in an apoE isoform–dependent manner. (**A** and **B**) Confocal image of IBA1-labeled microglia (green) and BACE-labeled neuritic dystrophy (magenta) costained around X34^+^ plaques (blue) in ipsi- (**A**) and contralateral (**B**) cortices from APPPS1:E3 and APPPS1:E4 mice from the NS or SD groups (*n* = 13–15 per group). Scale bars: 20 μm. (**C**–**H**) Quantification of the number of microglia surrounding plaques in the ipsi- and contralateral cortices (**C** and **D**, respectively), hippocampi (**E** and **F**, respectively), and thalami (**G** and **H**, respectively) of AD-tau–injected APPPS1:E3 and APPPS1:E4 mice. (**I**–**N**) Quantification of the percentage of BACE1^+^ voxels within 15 μm of plaques in the ipsi- and contralateral cortices (**I** and **J**, respectively), hippocampi (**K** and **L**, respectively), and thalami (**M** and **N**, respectively) of AD-tau–injected APPPS1:E3 and APPPS1:E4 mice. Data are presented as the mean ± SEM. Significance was determined by 3-way ANOVA with Šidák’s multiple-comparison test (sex, apoE genotype, and sleep condition). *P < 0.05 and **P < 0.01. See also Supplemental Table 1.

Given that previous studies found that microglial activation around plaques regulates the amount of neuritic dystrophy and Aβ-induced NP-tau seeding and spreading, we investigated whether microgliosis around plaques is affected by SD in the presence of Aβ-induced tau seeding and spreading (Figure 6, A and B). We observed significantly decreased IBA1^+^ microglial clustering around plaques in both ipsilateral and contralateral quantified brain regions of AD-tau–injected, sleep-deprived APPPS1:E4 mice compared with the NS group (Figure 6, C–H). Since previous research from our laboratory showed a strong correlation between the amount of BACE1^+^ neuritic dystrophy around plaques and NP-tau pathology (25), we next investigated whether chronic SD in AD-tau–injected APPPS1 mice could facilitate the accumulation of dystrophic neurites around plaques in an apoE-dependent manner. Swollen, dystrophic neural processes that contain aggregated, phosphorylated tau (p-tau) surrounding Aβ plaque deposits are a key feature of NP-tau pathology. Therefore, we quantified BACE1^+^ neuritic dystrophy formation around plaques by confocal imaging and found that chronic SD induced a significant increase in neuritic dystrophy, particularly in the cortex, in the presence of APOE4, but not APOE3, as compared with the NS groups (Figure 6, I–N).

### SD decreases perivascular polarization and mRNA and protein expression of aquaporin-4 in the presence of apoE4 but not apoE3.

**Figure 7:**
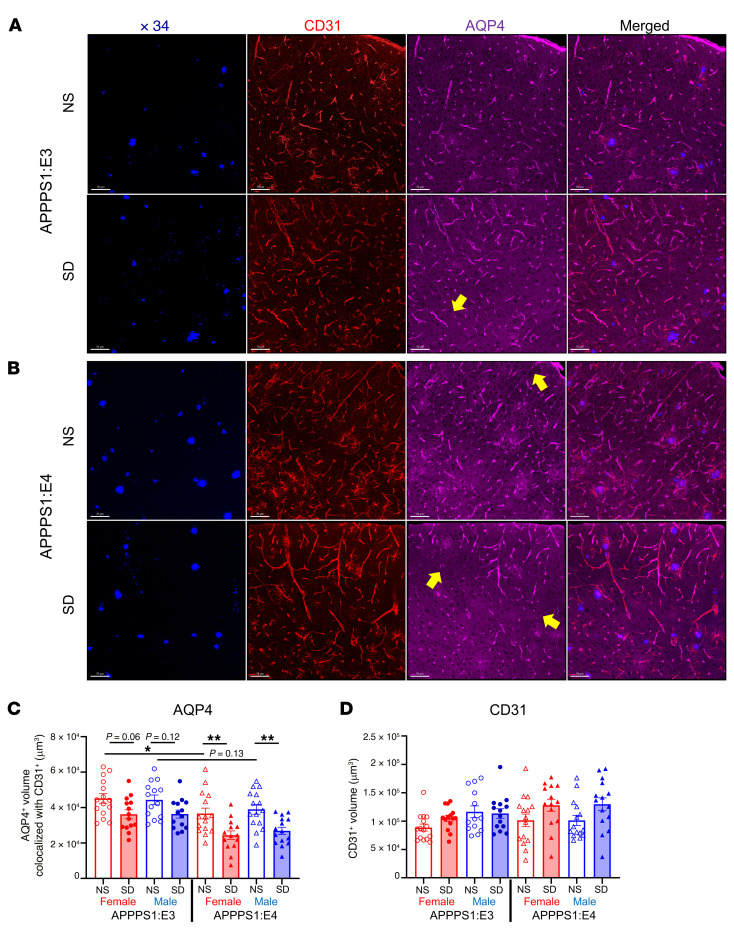
SD decreases perivascular polarization of AQP4 in an apoE isoform–dependent manner. (**A** and **B**) Confocal image of CD31-labeled blood vessel (red) and AQP4-labeled water channel AQP4 (magenta) costained with X34^+^ plaques (blue) in cortex from APPPS1:E3 (**A**) and APPPS1:E4 (**B**) mice from the NS and SD groups (*n* = 13–15 per group). Scale bars: 70 μm. Yellow arrows indicate a significant decrease in polarization of AQP4 around blood vessels. (**C**) Quantification of colocalized AQP4 and CD31 volumes in cortex from APPPS1:E3 and APPPS1:E4 mice. (**D**) Quantification of volume of CD31^+^ voxels in cortex from APPPS1:E3 and APPPS1:E4 mice. Data are presented as the mean ± SEM. Significance was determined by 3-way ANOVA with Sidak’s multiple-comparison test (sex, apoE genotype, and sleep condition). *P < 0.05 and **P < 0.01. See also Supplemental Table 1.

**Figure 8:**
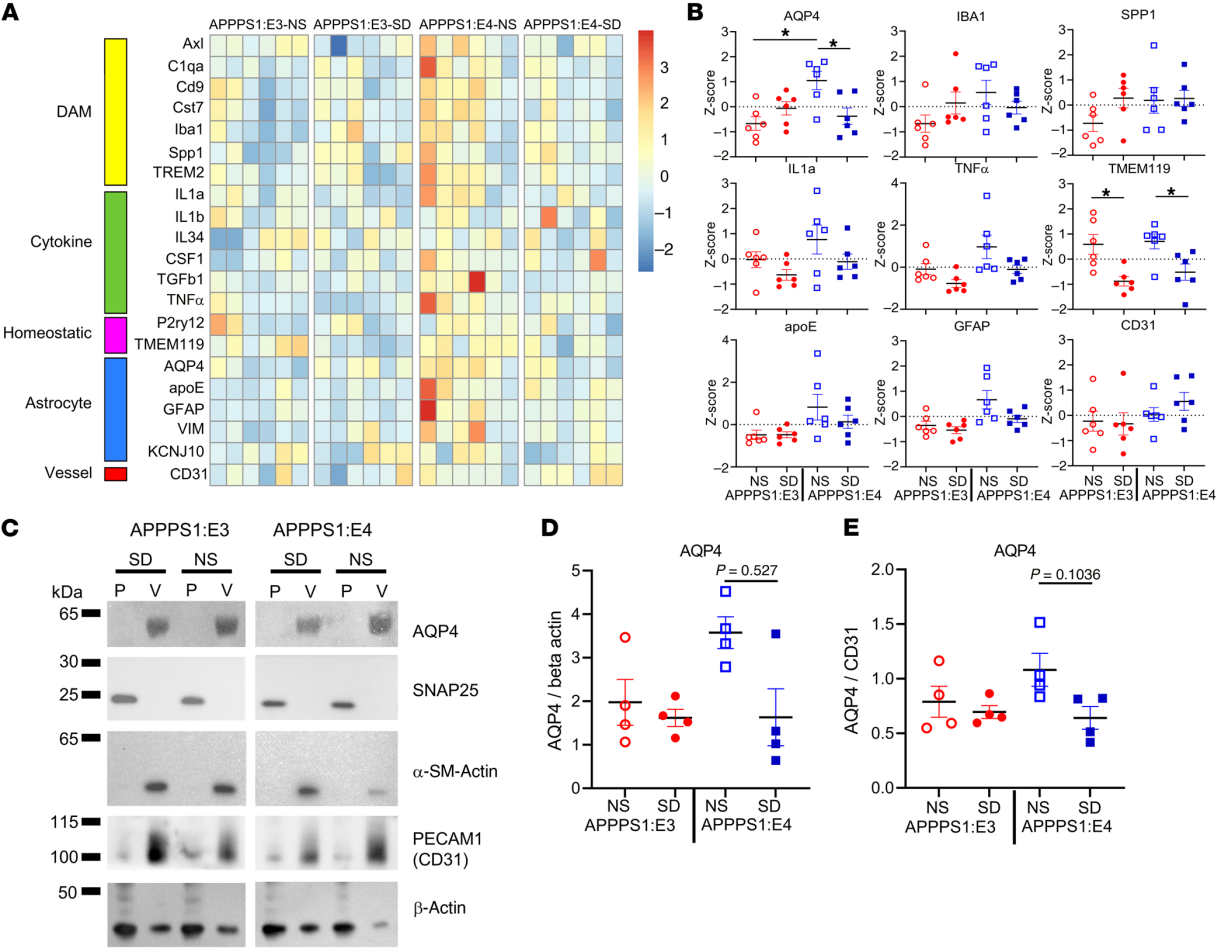
SD affects *AQP4* gene and protein expression in an apoE isoform–dependent manner. (**A**) Heatmap analysis of bulk RNA in cortices from APPPS1:E3 and APPPS1:E4 male mice that were subjected to NS or SD, generated by hierarchical gene clustering based on groups (*n* = 6 per group). (**B**) Selected heatmap analysis results from each cluster. Heatmap *z* scores were calculated for each gene and plotted instead of the normalized expression values. (**C**) Western blot images of AQP4, SNAP25 (neuronal marker, mainly detected in the parenchyma fraction), α-SMA (vascular marker), CD31, and the β-actin compartment from single mouse brain hemispheres (half-cerebral cortex) from APPPS1:E3 and APPPS1:E4 male mice that were subjected to NS or SD (*n* = 4 per group). V, vessel fraction; P, parenchyma fraction. (**D** and **E**) Quantitative analysis of AQP4 expression levels after normalization to β-actin (**D**) or CD31 (**E**). Data are presented as the mean ± SEM. Significance was determined by 2-way ANOVA followed by a Tukey’s post hoc test (apoE genotype and sleep condition) (**B**, **D**, and **E**). *P < 0.05. See also Supplemental Table 1.

We found that chronic SD significantly increased Aβ plaque deposition, fibrillar Aβ plaques, and Aβ-induced NP-tau seeding and spreading in an apoE isoform–dependent manner. The specific effect of SD only in APPPS1:E4, but not APPPS1:E3, mice may have been due to the observed decrease in microglial clustering surrounding plaques in the presence of E4 but not E3 following SD. However, we wanted to explore whether additional mechanisms contributed to this phenomenon. Previous work in our laboratory showed that human apoE isoforms differentially regulate the clearance of Aβ in brain ISF of PDAPP/ApoE mice, a mouse model of Aβ amyloidosis (12). Another potential mechanism underlying the differential effects of SD in the presence of E4 could be that there are apoE-dependent differences in the clearance of ISF Aβ (28–31). The aquaporin-4 (AQP4) water channel is a major driver of glymphatic clearance and facilitates the removal of extracellular Aβ and tau from the brain ISF to the CSF (30, 31). To determine whether chronic SD in APPPS1:E4, but not APPPS1:E3, mice induces the disruption of perivascular polarization of AQP4 that could influence ISF Aβ and subsequent plaque pathology, we performed confocal analysis and quantified the volume of perivascular AQP4 surrounding platelet endothelial cell adhesion molecule (PECAM-1), also known as cluster of differentiation 31 (CD31), blood vessels (Figure 7, A and B). Interestingly, we found that chronic SD significantly decreased the volume of perivascular polarized AQP4 in AD-tau–injected APPPS1:E4 mice as compared with the NS group (Figure 7C). We observed a nonstatistically significant trend toward a decreased volume of perivascular polarized AQP4 in SD-APPPS1:E3 mice compared with the NS group (Figure 7C). We did not observe significant changes in the volume of CD31^+^ blood vessels in either APPPS1:E3 or APPPS1:E4 mice (Figure 7D). After observing a decrease in perivascular polarized AQP4 in sleep-deprived experimental mice, we decided to investigate whether chronic SD affects the mRNA and protein levels of AQP4 in an apoE-dependent manner. Interestingly, *Aqp4* mRNA expression was higher in the NS APPPS1:E4 mice than in the APPPS1:E3 mice (Figure 8B). SD resulted in decreased *Aqp4* gene expression in the presence of apoE4, but not apoE3, relative to expression levels in NS controls, consistent with the results of the reduced perivascular polarization of AQP4 (Figure 8B). Simultaneously, we also investigated other gene expression profiles associated with disease-associated microglia (DAM), neuroinflammation (cytokines), microglial homeostasis, astrocytes, and blood vessels (Figure 8A). Evaluation of several inflammatory cytokine genes revealed no significant decrease in proinflammatory mediators such as IL-1α, IL-1β, TNF-α, and TGF-β in both SD-APPPS1:E3 and SD-APPPS1:E4 mice. Chronic SD significantly decreased the expression of homeostatic microglial genes such as *P2ry12* and *Tmem119* in both APPPS1:E3 and APPPS1:E4 mice. After confirming decreased expression of the *Aqp4* gene in sleep-deprived APPPS1:E4 mice, we next investigated whether chronic SD decreases the protein levels of AQP4 in an apoE isoform–dependent manner. Using a recently developed protocol (32) to separate the vascular compartment from brain parenchyma to study the entire vascular niche, including AQP4 protein, we performed purification of vessels from the cortex followed by Western blotting (Figure 8C). Vascular smooth muscle cell–specific α smooth muscle actin (α-SMA) was heavily enriched in the vessel fraction (V) (Figure 8C). By contrast, the neuronal synaptosomal-associated protein 25 (SNAP25) was mainly enriched in the parenchymal fraction (P) (Figure 8C). AQP4 was enriched in the vessel fraction, indicating attachment of perivascular astrocyte endfeet processes to the vessel fraction. Interestingly, baseline levels of AQP4 were high in APPPS1:E4 mice and were significantly decreased after SD (Figure 8, D and E). The abnormalities in AQP4 polarization and expression levels induced by SD in APPPS1:E4 mice resulted in disrupted astrocyte function as well as altered astrocyte clustering around plaques (Figure 3, C and E).

### Aβ deposition and peri-plaque NP-tau pathology significantly affects sleep behaviors in the presence of APOE4 but not APOE3.

**Figure 9:**
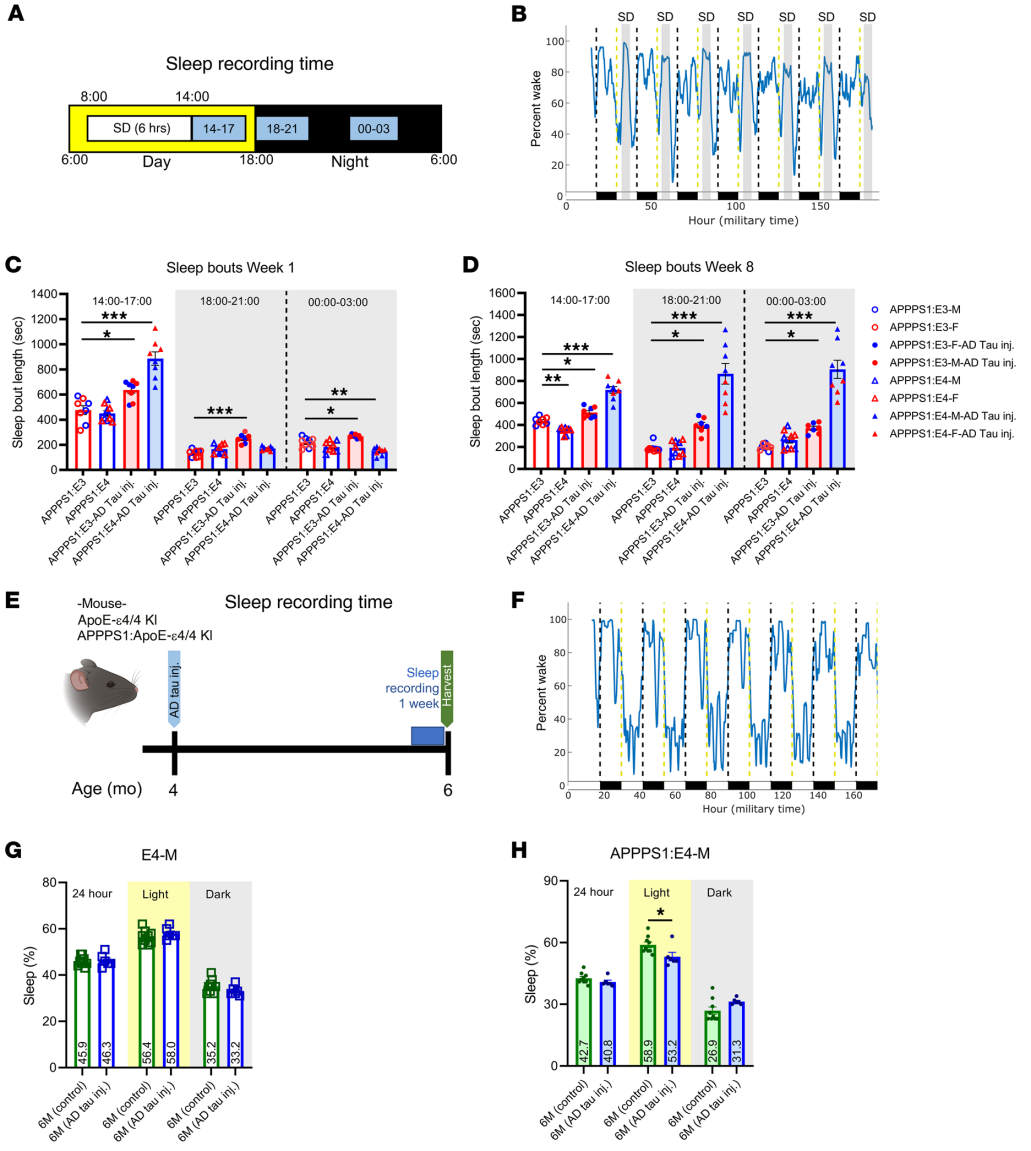
Aβ deposition and peri-plaque NP-tau pathology significantly affect sleep behaviors in the presence of APOE4 but not APOE3. (**A**) Schematic of the experimental design. Sleep-wake recording data were analyzed at 3 time points (14–17, 18–21, and 00–03) to investigate sleep rebound behaviors of APPPS1:E3 and APPPS1:E4 mice after SD (*n* = 3–6 per group). (**B**) Representative percentage wake plot in the SD condition. (**C** and **D**) Average sleep bout length for each group for 1400–1700, 1800–2100, and 0000–0300 hours for the first week of SD (**C**) and at week 8 of SD (**D**). F, female; M, male. (**E**) Schematic of the experimental design (*n* = 6–9 per group). (**F**) Representative percentage wake plot for the NS condition. (**G** and **H**) Average sleep percentage for each group for a 24-hour period, the light phase, and the dark phase in APPPS1:E4 male mice (**G**) and APPPS1:E4 male mice (**H**). 6M (control): *n* = 9; 6M (AD-tau inj.): *n* = 6. Data are presented as the mean ± SEM. Significance was determined by 3-way ANOVA with Šidák’s multiple-comparison test (sex, apoE genotype, and AD-tau injection) or Student’s *t* test (6M (control) versus 6M (AD-tau inj.)). *P < 0.05, **P < 0.01, and ***P < 0.001. See also Supplemental Table 1.

Our previous study showed that Aβ plaque deposition disrupts the sleep-wake cycle and diurnal fluctuation of Aβ in APPPS1 mice (33). Since we observed that chronic SD increased Aβ plaque deposition and Aβ-induced NP-tau seeding and spreading in APPPS1:E4 but not E3 mice, we hypothesized that chronic SD could induce significant changes in sleep rebound behavior in the presence of APOE4 but not APOE3, corresponding to the increased Aβ deposition and peri-plaque NP-tau pathology. Sleep rebound behavior is an increase in the amount of sleep following SD. To determine whether sleep rebound behavior in APPPS1:E4 mice but not APPPS1:E3 mice is significantly affected in response to chronic SD, we performed PiezoSleep recording with chronic SD treatment using a piezo sensor lining the bottom of the sleep fragmentation chamber (Figure 9, A and B). We also investigated the sleep rebound behavior of AD-tau–injected APPPS1:E3 and APPPS1:E4 mice. We selected and analyzed 3 time points (24-hour military time): 1400–1700 hours (right after SD); 1800–2100 hours (right after dark onset); and 2400 to 0300 hours (after midnight) after the SD treatment time (0800–1400 hours). We also compared sleep rebound behaviors between the first week and the last week (eighth week) of the SD treatment period (Figure 9A). We observed strong trends toward decreased sleep percentages in both AD-tau–injected and non–AD-tau–injected APPPS1:E4 mice after the SD time (1400–1700 hours) after 8 weeks of SD treatment compared with the first week of SD. In addition, we observed strong trends toward increased sleep percentages (1400–1700 hours) in both AD-tau–injected and non–AD-tau–injected APPPS1:E3 mice after 8 weeks of SD treatment compared with the first week of SD (Supplemental Figure 4, A and B). We also observed strong trends toward increased sleep percentages during the dark phase (1800–2100 and 0000–0300 hours, respectively) in all experimental groups after 8 weeks of SD treatment compared with the first week of SD (Supplemental Figure 4, A and B). Interestingly, we observed significantly increased sleep bout lengths during the dark phase (1800–2100 and 0000–0300 hours, respectively) in AD-tau–injected APPPS1:E4 but not APPPS1:E3 mice after 7 weeks of SD treatment compared with the first week of SD (Figure 9, C and D). We did not observe significant changes in sleep bout lengths in the non–AD-tau–injected mouse groups (Figure 9, C and D). This suggests that significantly increased Aβ plaque deposition and NP-tau pathology in APPPS1:E4 mice may have induced decreased neuronal/synaptic function controlling sleep and wake states because average sleep bout lengths should not be high during the dark phase. Furthermore, we observed significantly lower wake bout lengths during the dark phase (1800–2100 hours) in both AD-tau–injected and noninjected APPPS1:E4 mice compared with APPPS1:E3 mice during the first week of SD (Supplemental Figure 4, C and D). However, the sleep behaviors of APPPS1:E4 and APPPS1:E3 mice were similar during the last week of the SD treatment period (Supplemental Figure 4, C and D). Overall, these findings suggest that the *APOE* genotype–dependent effect of SD may have led to different changes in sleep rebound behavior in the experimental mice.

After observing that sleep rebound behaviors in AD-tau–injected APPPS1:E4 mice were significantly affected, especially in male APPPS1:E4 mice, we next assessed whether Aβ-induced NP-tau pathology in APPPS1:E4 male mice exacerbates their sleep quality and quantity under non-SD conditions compared with noninjected APPPS1:E4 male mice. Using the PiezoSleep recording system (Figure 9, E and F), we investigated sleep percentages, sleep bout lengths, and wake bout lengths in AD-tau–injected APPPS1:E4 male mice at 6 months of age as well as noninjected APPPS1:E4 male mice at the same age, which served as the control group (Figure 9, G and H, and Supplemental Figure 5, A–D). To further investigate the effects of AD-tau injection, we also recorded APOE4-knockin male mice under the same conditions. Similar to our prior study, which showed disrupted sleep phenotypes depending on the level of Aβ deposition (33), we also observed that increased Aβ deposition in APPPS1:E4 male mice with AD-tau injection at 6 months of age was associated with decreased overall sleep percentages and increased sleep bout lengths as compared with APOE4-knockin (apoE4) male mice (Figure 9, G and H, and Supplemental Figure 5A and B). Interestingly, we noted a significant decrease in sleep percentages in AD-tau–injected APPPS1:E4 mice during the light phase compared with their control group (non–AD-tau–injected APPPS1:E4 mice) (Figure 9H). However, we did not see any changes in sleep percentages in the APOE4 mouse groups (Figure 9G). Additionally, we observed significantly increased sleep bout lengths in AD-tau–injected APPPS1:E4 mice compared with their control group (Supplemental Figure 5B). However, the wake bout length for AD-tau–injected APPPS1:E4 mice was not significantly changed compared with their control group except during the light phase (Supplemental Figure 5D). Last, we observed increased sleep bout lengths throughout a 24-hour day and during the light phase in AD-tau–injected APOE4 male mice compared with their control group (Supplemental Figure 5A). We did not see any changes in wake bout lengths in the APOE4 mouse groups (Supplemental Figure 5C). Taken together, these results suggest that tau pathology induced by AD-tau injection may differentially affect sleep behavior in mice, in the presence or absence of Aβ.

Finally, we assessed the sleep-wake regularity of several parameters such as mean sleep percentage, sleep bout length, and sleep fragmentation across 24-hour periods in APPPS1:E3 and APPPS1:E4 mice at 6 months of age under non-SD conditions, with the goal of providing further insight into how APOE4 may modify sleep-wake behavior. First, using the PiezoSleep recording system, we analyzed the mean sleep percentage. We found that both APPPS1:E4 male and female mice had significantly decreased mean sleep percentages across 24-hour periods compared with APPPS1:E3 male and female mice, respectively (Supplemental Figure 6A). We also found that the mean sleep bout length was significantly decreased in APPPS1:E4 male mice versus APPPS1:E3 male mice (Supplemental Figure 6B). There was no difference between mean sleep bout length in APPPS1:E4 female mice versus APPPS1:E3 female mice (Supplemental Figure 6B). Second, after exporting activity data from the PiezoSleep, which recorded the raw data, and using Clocklab software, we assessed sleep fragmentation across 24-hour periods by analyzing the intradaily variability (IV) results. We observed that APPPS1:E4 male mice had significantly more sleep fragmentation throughout 24-hour periods compared with APPPS1:E3 male mice (Supplemental Figure 6C). We did not observe significant differences in IV between the female mice in the presence of APOE4 or APOE3 (Supplemental Figure 6C). Third, we also analyzed the interdaily stability (IS) results to check inter-day regularity in sleep and wake parameters. We did not observe statistically significant differences in IS between the APPPS1:E3 and APPPS1:E4 mice (Supplemental Figure 6D). Last, we observed no significant differences in the amplitude of activity between APPPS1:E3 and APPPS1:E4 mice (Supplemental Figure 6E).

## Discussion

Sleep is essential to many biological functions, including memory consolidation, cognitive performance, nerve cell communication, and neurotoxin clearance (28, 34, 35). Chronic poor sleep affects cognitive function and results in functional impairment in patients with AD (21, 36). Despite the importance of sleep and the prevalence of sleep disruptions observed across multiple neurodegenerative diseases, especially AD, little is known about the interactions between sleep, *APOE* genotype, and AD pathology. In this study, we show that chronic SD markedly affected AD pathology in an apoE isoform–dependent manner. First, as sleep loss induces increased Aβ deposition (7, 8), we hypothesized that *APOE4* might synergize with chronic SD to accelerate Aβ plaque deposition. Using APPPS1 mice expressing human APOE3 or APOE4 in the absence of murine apoE, we confirmed that chronic SD in APPPS1 mice significantly increased (*P* < 0.01) both total and fibrillar Aβ plaque deposition but only in the presence of APOE4 and not APOE3.

We hypothesized that the differential increase in Aβ plaque deposition might be mediated by SD-induced changes in plaque-associated microglia and/or astrocytes in an apoE isoform–dependent fashion. Indeed, we found that chronic SD decreased both astrocyte, and particularly microglial, clustering around plaques, but only in the presence of APOE4 and not APOE3. Astrocyte clustering around plaques in the cortex and hippocampus showed strong trends toward decreased clustering in the presence of APOE4 but not APOE3. However, astrocyte clustering around plaques in the thalamus showed a significant increase in the presence of APOE4 but not APOE3. The thalamus plays a vital role in sleep-wake regulation; it becomes quiet during most stages of sleep and then becomes active during the wakened state (37). Chronic SD may affect the reactivity of astrocytes to Aβ plaques in the thalamus due to increased wakefulness. Surprisingly, we found that chronic SD significantly decreased (*P* < 0.05) the number of microglia around plaques in the presence of APOE4 but not APOE3. A previous study showed that SD promotes microglial activation in the mouse cerebral cortex in response to increased neuronal activity to remove heavily used synapses that have accumulated presynaptic components (24). In regard to the role of APOE4 in microglia, a recent in vitro study showed that microglia in the presence of APOE4, but not APOE3, disrupts the coordinated activity of neuronal ensembles because apoE4 induces a lipid-laden state that impairs the microglial response to neuronal activity (38). Our findings demonstrate that APOE4 might synergize with chronic SD to impair the microglial response to the accumulation of Aβ, which could accelerate Aβ plaque deposition via impairment of microglial phagocytosis of plaques. In addition to increased Aβ plaque deposition, previous studies suggested the idea that microglia restrict the formation of peri-plaque neuritic dystrophy (25, 39–41). Consistent with this idea, we also found that the decreased microglia clustering around plaques due to chronic SD in APPPS1:E4 mice induced significant increases in neuritic dystrophy around plaques (*P* < 0.05). Future studies should explore whether selective removal of microglial or astrocytic *APOE4* rescues the microglial response to changes in soluble or insoluble Aβ in sleep-deprived APPPS1:E4 mouse brain to determine whether this effect of ApoE4 is cell autonomous.

In humans, the progression of tau pathology into the limbic cortex and neocortex is driven by the presence and progression of Aβ plaque deposition (42–44). The presence of Aβ plaques promotes local tau seeding in neuritic dystrophy, which leads to the spreading and formation of p-tau in NP-tau aggregates and neurofibrillary tangles (NFTs) in mice (26). Therefore, using the previously described AD-tau injection model (25–27, 45), we investigated whether chronic SD in APPPS1:E4, but not APPPS1:E3, mice significantly facilitates NP-tau pathology by exacerbating tau seeding and spreading. We confirmed that NP-tau pathology around the Aβ plaques in APPPS1:E4, but not APPPS1:E3, mice that represent both seeding and spreading were also significantly increased by chronic SD. Importantly, we demonstrated that NP-tau seeding and spreading induced by chronic SD was associated with decreased microglia clustering and increased neuritic dystrophy in the presence of APOE4. Further analysis revealed a trend toward or significantly reduced expression of microglial markers such as *Cst7*, *Iba1*, *Trem2*, *P2ry12*, and *Tmem119* in the presence of APOE4 but not APOE3. This might be due to synergism of APOE4 with chronic SD to induce microglia to assume a more static, lipid-accumulated state, thereby rendering the microglia unable to appropriately respond to the progression of AD pathology (38, 46). However, we only treated mice with chronic SD from 4–6 months of age. It is certainly possible that SD could exacerbate Aβ and tau pathology in the presence of APOE3 if the mice were exposed to a longer period of SD and were followed for longer than 2 months. Further experiments will be required to address this issue.

Our study demonstrates that the *APOE4* genotype synergizes with SD to exacerbate Aβ plaque deposition and NP-tau pathology along with decreased microglia clustering and increased formation of dystrophic neurites around plaques. On the basis of our findings, we investigated whether the *APOE* genotype also alters the cellular elements important in the glymphatic system, which appears to be involved in the clearance of extracellular molecule species in the brain (47). Several studies suggest that the glymphatic clearance system plays a role in the net clearance of Aβ and tau from the brain (28–31). Our previous research showed that the *APOE* genotype contributes to AD risk by differentially regulating clearance of Aβ from the brain (12, 22). Additionally, extended wakefulness due to chronic SD leads to a significant increase in the release of soluble Aβ and tau in the ISF as a result of increased neuronal activity (7, 9). Therefore, we hypothesized that the *APOE4* genotype synergizes with chronic SD to induce the disruption of perivascular polarization of AQP4, a major driver of glymphatic clearance that facilitates the removal of extracellular soluble Aβ and tau from the brain parenchyma (30, 31). Interestingly, we found that chronic SD significantly decreased (*P* < 0.01) the volume of perivascular polarized AQP4 staining in the cortex of AD-tau–injected APPPS1:E4 mice. The decreased polarization of AQP4 is strongly associated with less effective clearance of the AD-related proteins from the brain parenchyma (31, 48, 49). This is further supported by our data showing that chronic SD significantly decreased (*P* < 0.05) mRNA and protein expression of AQP4 in APPPS1:E4 mice. Our findings suggest that chronic SD with *APOE4* genotype synergizes to impair AQP4 polarization, which may contribute to exacerbation of Aβ plaque deposition and Aβ-associated tau seeding and spreading via less functional glymphatic clearance. Future studies will explore how the *APOE* genotype coupled with chronic SD affects perivascular polarization of AQP4 on astrocytic endfeet in relation to astrocytic homeostasis. It is also possible that chronic SD, by influencing microglia/myeloid function, worsens glymphatic function. A recent study showed that perivascular macrophages regulate glymphatic function (50). It was found that parenchymal border macrophages (PBMs) regulate CSF flow dynamics. In the homeostatic brain, the major populations of macrophages are microglia, which are located in the brain parenchyma, and PBMs, which are located in the leptomeningeal and perivascular spaces along the vasculature of the brain (50). As we found that chronic SD significantly decreased microglial clustering around plaques, chronic SD with the APOE4 genotype may synergize to impair the function of PBMs in regulating CSF flow. This might then affect astrocytes around blood vessels and impact the polarization of AQP4. Future studies should explore how chronic SD coupled with the *APOE* genotype affects CSF flow dynamics via assessment of PBM function. The sleep-wake cycle regulates soluble Aβ and tau levels in the brain (7, 9). Both mouse models of β-amyloidosis and tauopathy have shown disrupted sleep phenotypes, which correlated with increases in each AD pathology (33, 51). Therefore, we hypothesized that significantly increased NP-tau pathology in sleep-deprived, AD-tau–injected APPPS1:E4 mice would further disrupt their sleep rebound behavior, quantity, and quality compared with control APPPS1:E4 mice. We found significantly increased sleep rebound behavior (*P* < 0.05) during the dark phase (1800–2100 and 0000–0300 hours, respectively) in AD-tau–injected APPPS1:E4 but not in APPPS1:E3 mice under SD conditions using the PiezoSleep recording system. Sleep rebound behavior after SD might be beneficial to recovery from sleep deficiency. However, we observed that the chronically sleep-deprived AD-tau–injected APPPS1:E4 mice showed fewer locomotor behaviors during the dark phase (active time for mice). We also observed some sex differences in sleep bout lengths in our study. Specifically, we observed that AD-tau–injected APPPS1:E4 male mice had significantly longer sleep bout lengths during the dark phase (1800–2100 and 0000–0300 hours, respectively) than did female mice after 7 weeks of SD treatment compared with the first week of SD (Figure 9D). As we observed no sex-based differences in the amyloid deposition in the amyloid-depositing mice we used, it is unclear if these changes in sleep behavior between male and female mice are meaningful with regard to pathology. However, in other amyloid-depositing mice such as 5XFAD, the females develop more amyloid pathology than do males, and such changes in sleep could be relevant to those changes (52). In humans, sleep and sleep disturbances differ between women and men (53). For instance, insomnia is more prevalent in women than in men. In animal studies in relation to age-related changes in sleep and sleep rebound behavior, one study using C57BL/6J mice reported that male mice at 8–10 weeks of age spent 25% more time in non–rapid eye movement (NREM) sleep (i.e., “deep sleep”) than did females over a 24-hour period, particularly in the dark phase. Also, this study reported that recovery of sleep after 6 hours of SD was similar between the sexes in terms of absolute amounts of sleep (54). Another study that used slightly older C57BL/6J mice (12 weeks old) reported that male mice spent more time in NREM sleep than did female mice over the 24-hour period, but homeostatic sleep pressure accumulated faster during the 8 hours of SD treatment, and REM sleep recovery after SD was more efficient in male mice than in female mice (55). Finally, a study assessed older but not-yet middle-aged C57BL/6J mice (4–6 months old) and found only slight sex differences in baseline sleep patterns and sleep recovery after 6 hours of SD, whereas much greater sex differences were observed in sleep responses after 1 hour of restraint stress at the beginning of the daily light phase (56). Based on our results, the sex differences in sleep rebound behaviors after 7 weeks of chronic SD might be exacerbated by the presence of APOE4 and NP-tau pathology induced by AD-tau injection. Furthermore, AD-tau–injected APPPS1:E4 mice under normal sleep conditions showed markedly decreased sleep percentages throughout a 24-hour day and during the dark phase and also a trend toward decreased wake bout lengths during the dark phase as compared with the control groups. In addition, we observed increased sleep bout lengths during the light phase and decreased wake bout lengths during the dark phase in AD-tau–injected apoE4 male mice compared with their control group (noninjected control group). One observation that might explain some of these findings is that AD-tau injection induced the formation of NFTs in the supramammillary nucleus (SuM), which is synaptically connected to the dentate gyrus (one of the AD-tau injection sites) and is well known as a brain region that controls wakefulness (data not shown). Moreover, we observed more NFTs in the SuM in APOE4 mice than in APOE3 mice (data not shown). These findings suggest that tau pathology induced by AD-tau injection and/or Aβ plaque deposition in the presence of APOE4 may synergize to weaken sleep-wake regulation and especially promote wakefulness. Further studies are needed to explore the role of APOE4 in the synaptic spreading of tau and the regulation of the sleep-wake cycle. Because the piezoelectric system we used for this study relies on breathing patterns to distinguish between sleep and wake states, slight movements during sleep states may have been misinterpreted as wakefulness. Likewise, during the wake period, there may have been moments of almost no movement and breathing patterns that happened to mimic sleep, causing some overscoring of sleep. However, in a wide variety of settings of PiezoSleep recording, both sensitivity and specificity for scoring sleep versus wake have consistently been above 90% relative to the “gold standard” of electroencephalography/electromyography (EEG/EMG).

This study demonstrates that *APOE4*, one of the strongest genetic risk factors for AD, exacerbated sleep disruption, accelerating Aβ plaque deposition and Aβ-mediated tau pathology. Our results suggest that a primary mechanism may be the synergistic effects of sleep disruption and APOE4, resulting in an impaired response by microglia and astrocytes to amyloid deposition. Approximately 25%–66% of patients with AD experience sleep disturbances, which also occur during the preclinical stage of AD (4). Further translational studies are required to explore whether improving sleep quality and quantity in individuals who are *APOE4* positive can delay the progression of AD pathology during the preclinical and early clinical stages of AD. Taken together, these findings offer a better understanding of the relationship between the *APOE* genotype and sleep in Aβ and Aβ-linked tauopathy and suggest unique early therapeutic strategies to treat AD.

## Methods

### Animals.

The APPPS1-21 mouse model of β-amyloidosis–expressing human amyloid precursor protein (APP) with the Swedish mutation (KM670/671NL) and L166P-mutated human presenilin1 (PS1) under the control of a neuron-specific Thy1 promoter were a gift from M. Jucker (Hertie Institute for Clinical Brain Research, University of Tübingen, Tübingen, Germany). Human ApoE-knockin (apoE3^fl/fl^ and apoE4^fl/fl^ ) and ApoE-KO mice were generated as previously described (23). APPPS1-transgenic mice with different human *APOE* genotypes (*APOE*-ε3 or -ε4) were generated as previously described (23). Male and female mice were used in this study, unless otherwise specified. All mice were bred on a C57BL/6 background and housed in specific pathogen–free conditions. All mice were housed under the same 12-hour light/12-hour dark cycle with food and water available ad libitum. The room temperature was kept at around 22°C.

### Preparation of AD-tau aggregates from human AD brain tissue.

AD-tau was isolated as previously described from human AD brain with Braak stage VI tau pathology (25, 45, 57). The total protein concentration was estimated to be 21.1 μg/μL using the Micro BCA protein assay kit (23225; Thermo Fisher Scientific). A tau-specific sandwich ELISA measured the tau concentration at 5.4 μg/μL. Before injection, AD-tau was diluted to a final concentration of 0.4 μg/μL and sonicated in a water bath sonicator (Q700; QSonica) for 30 seconds at 65% amplitude at 4°C.

### Stereotactic intracerebral injections of AD-tau.

Four-month-old experimental mice were anesthetized using isoflurane, immobilized in a stereotactic frame (model 942; David Kopf Instruments), and unilaterally injected with 2 μg AD-tau (1 μg at each injection site) in the dentate gyrus (bregma: –2.5 mm; lateral: –2.0 mm; depth: –2.2 mm) and overlying cortex (bregma: –2.5 mm; lateral: –2.0 mm; depth: –1.0 mm) using a Hamilton syringe (Hamilton; syringe: 80265–1702RNR; needle:7803–07, type 4, 1.5 inch needle length, 60° angle) as previously described (25, 27, 45). Mice were allowed to recover on a heating pad and monitored for 72 hours after surgery.

### SD.

Mice were placed in automated sleep fragmentation chambers (model 80391; LaFayette Instruments) with corn cob bedding. An automated swipe bar inside of the sleep fragmentation chamber moved horizontally across the cage and was set at 30-second intervals for 6 hours a day, starting 2 hours after light onset over 8 weeks. The duration of SD was based on previous findings (7). Mice were monitored daily for signs of stress or health decline. Control (NS) mice were kept in their home cages in the same room.

### Measurement of sleep and wake states.

Sleep and wake states were determined using a noninvasive piezoelectric system (Signal Solutions). The piezoelectric system has been described in detail in previous publications (58, 59). Briefly, it is composed of a Plexiglass cage with a piezoelectric sensor film lining the bottom that detects pressure variations due to the movement of the experimental mice. Mice were individually housed in each piezosensor cage with fresh water and food available ad libitum and recorded without disturbance over a period of 6 days. The sleep-wake states were analyzed by SleepStats software (Signal Solutions). As noted in the Mang et al. study (59), the most common sleep bout lengths (when scoring every 4-second window or epoch) are bout lengths in the 32- to 60-second and 64- to 124-second range over a 24-hour period, which are nearly identical when using EEG/EMG or PiezoSleep. However, PiezoSleep does appear to somewhat overscore very short bouts, probably because of animal twitching or subtle movements during sleep that are not enough to alter EEG/EMG scoring, or, in some cases, other sources of “noise” that cause 1 or more 4-second windows to flip from sleep to wake for 1 or more 4-second windows. Therefore, we used 30-second epochs for scoring sleep bout lengths following the manufacturer’s default setting in the current version of SleepStats software. The most common sleep bout length using 30-second epochs is in the 400- to 600-second range over a 24-hour period. For the measurement of sleep rebound behavior, we used piezosensors with sleep fragmentation chambers. A specially designed metal part to protect the piezosensor (gift from Bruce O’Hara and Signal Solutions for testing the new setup) covered the piezosensor lining the bottom of the sleep fragmentation chamber. The piezosensor and the protective metal part were set up underneath fresh bedding. The same recording and analysis software described above was used. For the measurement of sleep-wake regularity (IV and IS), we used Clocklab software after exporting activity data from the original PiezoSleep raw recording data.

### Brain extraction and preparation.

All mice were perfused during the SD period, between 0800 and 1400 hours. Mice were anesthetized by intraperitoneal injection of pentobarbital (200 mg/kg). Blood samples were collected in EDTA-treated tubes prior to cardiac perfusion with 3 U/mL heparin in cold Dulbecco’s PBS. For 6-month-old AD-tau–injected or noninjected experimental mice, whole brains were carefully extracted and fixed in 4% paraformaldehyde for 48 hours before being transferred to 30% sucrose and stored at 4°C until they were sectioned. Brains were cut coronally into 30 μm sections on a freezing sliding microtome (SM1020R; Leica) and stored in cryoprotectant solution (0.2 M PBS, 15% sucrose, and 33% ethylene glycol) at –20°C until use. A small notch was placed on the left hemisphere with a clean razor blade to ensure identification of the ipsilateral injected side. For biochemical analysis, the right hemispheres of some noninjected experimental mice were dissected to isolate the cortex and the hippocampus, and the tissues were kept at –80°C until analyzed.

### Hippocampus.

For staining of the hippocampus for total Aβ (HJ3.4 biotinylated, anti–Aβ1-13, mouse monoclonal, 1:1,000, 2.81 μg/mL; generated in-house) and NP-tau (AT8 biotinylated, mouse monoclonal, 1:500, MN1020B; Thermo Fisher Scientific), sections were washed 3 times in TBS for 5 minutes and incubated in 0.3% hydrogen peroxide for 15 minutes. After washing, sections were blocked in 3% milk in TBS with 0.25% Triton X-100 for 30 minutes. Primary antibodies were diluted in 3% milk/Triton X-100, and the sections were incubated with the primary antibodies overnight at 4°C. The next day, sections were washed 3 times in TBS. For HJ3.4 and AT8 staining, after washing, sections were incubated in ABC Elite solution (PK-6100; VectaStain) for 1 hour, according to the manufacturer’s instructions. After washing, sections were developed in 3,39-diaminobenzidine (SK4103) solution (Vector Laboratories for HJ3.4B and D5905; MilliporeSigma for AT8B), washed, and mounted onto slides. After drying overnight, the slides were dehydrated in increasing ethanol concentrations followed by xylene and coverslipped using Cytoseal 60 (8310; Thermo Fisher Scientific). Slides were scanned on a NanoZoomer 2.0-HT system (Hamamatsu Photonics). Images were processed using NDP viewing software (Hamamatsu Photonics) and quantified with the use of Fiji software, version 2.1.0 (NIH). All areas were quantified in 2–3 sections (180 μm apart from each other) per mouse.

### Immunofluorescence.

For immunofluorescence (IF) staining, costaining was performed for (a) X34, BACE1, AT8, and IBA1; (b) X34, BACE1, and GFAP; or (c) X34, CD31, and AQP4. Fibrillar Aβ was stained by X34 dye (SML-1954, 1:5,000; MilliporeSigma), and BACE1 (ab108394, 1:500; Abcam), AT8 (MN1020B, 1:500; Thermo Fisher Scientific), IBA1 (ab5076, 1:500; Abcam), GFAP (53-9892-82, 1:500; Thermo Fisher Scientific), CD31 (MAB1398Z, 1:300; MilliporeSigma), and AQP4 (CL647-16473, 1:500; Proteintech) antibodies were used to evaluate peri-plaque pathologies. Free-floating sections were washed 3 times in PBS for 5 minutes each and then permeabilized in 0.25% Triton X-100 PBS (PBS-X) for 30 minutes. Tissue sections were then incubated in X34-0.1M NaOH for 20 minutes, washed in X34 buffer (40% EtOH in PBS), and then washed twice in PBS. Sections were incubated in blocking solution for 30 minutes (3% BSA, 3% normal donkey serum, and 0.1% PBS-X) before incubation in primary antibodies in blocking solution overnight at 4°C. The next day, sections were washed 3 times in PBS for 5 minutes each, placed in secondary antibodies for 2 hours (1:500; Thermo Fisher Scientific; for anti–Armenian hamster [for CD31], 127-295-099, 1:300; Jackson ImmunoResearch) at room temperature (RT), and then washed 3 times in PBS for 20 minutes each. Lipofuscin was quenched with 0.1% Sudan black in 70% ethanol through a 10-minute incubation, rinsed with 70% ethanol, and washed once in 0.02% PBS–Tween-20 for 5 minutes and then in PBS for 5 minutes. Sections were mounted, sealed in Fluoromount-G (0100-01; SouthernBiotech), and stored in the dark at 4°C until imaging.

### Confocal imaging and analyses.

IF images were acquired using a Leica Stellaris 5 confocal microscope and the Leica Application Suite X software (4.2.1.23810). Laser and detector settings were maintained for the acquisition of each immunostaining. For all analyses, at least 3 images were taken per brain region and slide using a 20× objective with 1× or 2× zoom, a 40× objective, and 1,024 × 1,024 resolution. Quantification of confocal images for AT8, BACE1, and GFAP volume around X34^+^ plaques was performed on a semiautomated platform using MATLAB and Imaris 9.5 software (Bitplane). To create surfaces of each stain based on a threshold applied to all images, X34^+^ surfaces were dilated by 15 μm and colocalized with various immunostained surfaces. For quantification of the number of plaque-associated IBA1^+^ microglia, a threshold was applied across all images to assign spots to each cell body or punctum. X34 surfaces were dilated to 15 μm, and spots were counted within the X34^+^ dilated surface. Any spots fully within or partially touching the extended surface were included in the analysis. For quantification of AQP4 volume colocalized on the vessel, CD31^+^ surfaces were dilated by 1 μm, and the AQP4^+^ volumes were quantified within the CD31^+^ dilated surface. All staining experiments were quantified by a blinded investigator.

### RNA extraction and gene expression analysis.

Frozen hippocampal tissue was weighed and homogenized in RNAase-free beaded tubes (REDE, Next Advance) in chloroform with TRIzol (Invitrogen). Samples were centrifuged for 15 minutes at 12,000*g* at 4°C, and the aqueous upper supernatant was transferred for RNA isolation with the RNeasy Plus Mini Kit (QIAGEN) following the manufacturer’s instructions. Gene expression analysis was performed using microarray in collaboration with the Genome Technology Access Core at Washington University. Using TaqMan probes probes (Thermo Fisher Scientific), relative gene expression was quantitatively measured using Fluidigm Biomark HD with integrated fluidic circuits.

### Brain vessel parenchyma fractionation and immunoblotting.

To analyze AQP4 levels colocalized with the vessel in the cortex specifically, brain vessels were separated from the parenchyma by a procedure previously described by Matthes et al. (32). This protocol was modified from the original one (60) and scaled down for use with 1 mouse brain hemisphere or less volume (see ref. 32 for more details). To extract protein, purified vessels were thawed by mixing with 100 μL RIPA buffer containing 10 mM Tris (pH 8.0), 1 mM EDTA, 1% Triton X-100, 0.1% sodium deoxycholate, 0.1% SDS, 140 mM NaCl, protease inhibitor (cOmplete, Roche), and phosphatase inhibitor cocktails (PhosSTOP, Roche) and then homogenized using a pestle with at least 20 strokes (32). Total protein (3 μg) of the vessel-enriched fraction and/or parenchymal fraction was subjected to SDS-PAGE and Western blotting. Samples were loaded onto a 4%–12% NuPAGE Bis-Tris gel (NP0329; Thermo Fisher Scientific) and transferred onto a PVDF membrane. Membranes were blocked in 5% BSA-TBS with 0.02% Tween-20 for 1 hour and incubated in primary antibody solution (AQP4, ab9512, 1:1,000, Abcam; SNAP25, 60159-1,1:5,000, Proteintech; α-SMA, 19245, 1:1,000, Cell Signaling Technology; CD31, 28083-1, Proteintech; β-actin, 81115-1-RR, Proteintech) diluted in 5% BSA-TBS with 0.02% Tween-20 overnight with shaking at 4°C. The next day, after washing, secondary antibodies were applied for 1 hour and shaken at RT. Membranes were washed 3 times for 5 minutes, developed using Lumigen CL Ultra Western Blotting HRP Substrate (TMA-100; Lumigen), and imaged using a ChemiDoc imaging system. Blots were converted to grayscale, and densitometric analysis was performed using ImageJ (NIH).

### Statistics.

Unless otherwise stated, all data are presented as the mean ± SEM. GraphPad Prism 8.0.0 (GraphPad Software) was used to generate data plots and to perform all statistical analyses. A Student’s *t* test or 2- or 3-way ANOVA multiple-comparison test was used to assess significance between more than 2 groups. A *P* value of less than 0.05 was considered statistically significant. See also Supplemental Table 1 for the statistical analysis details.

### Study approval.

All animal procedures and protocols were approved by the Animal Studies Committee at Washington University School of Medicine, St. Louis, Missouri, USA.

## Author contributions

CW and DMH designed the study. CW, AN, MRS, CL, SP, ACM, ESM, and JDU performed the experiments and analyzed the data. CW and DMH wrote the manuscript. All authors discussed the results and commented on the manuscript.

## Supplementary Material

Supplemental data

## Version 1 06/06/2023

In-Press Preview

## Version 2 07/17/2023

Electronic publication

## References

[1] Van Erum J (2018). Sleep and Alzheimer’s disease: A pivotal role for the suprachiasmatic nucleus. Sleep Med Rev.

[2] Selkoe DJ (1997). Alzheimer’s disease: genotypes, phenotypes, and treatments. Science.

[3] Bianchetti A (1995). Predictors of mortality and institutionalization in Alzheimer disease patients 1 year after discharge from an Alzheimer dementia unit. Dementia.

[4] Guarnieri B (2012). Prevalence of sleep disturbances in mild cognitive impairment and dementing disorders: a multicenter Italian clinical cross-sectional study on 431 patients. Dement Geriatr Cogn Disord.

[5] Okuda S (2019). Association between sleep disturbance in Alzheimer’s disease patients and burden on and health status of their caregivers. J Neurol.

[6] Ju YE (2013). Sleep quality and preclinical Alzheimer disease. JAMA Neurol.

[7] Kang JE (2009). Amyloid-beta dynamics are regulated by orexin and the sleep-wake cycle. Science.

[8] Roh JH (2014). Potential role of orexin and sleep modulation in the pathogenesis of Alzheimer’s disease. J Exp Med.

[9] Holth JK (2019). The sleep-wake cycle regulates brain interstitial fluid tau in mice and CSF tau in humans. Science.

[10] Mahley RW (2000). Apolipoprotein E: far more than a lipid transport protein. Annu Rev Genomics Hum Genet.

[11] Kim J (2009). The role of apolipoprotein E in Alzheimer’s disease. Neuron.

[12] Castellano JM (2011). Human apoE isoforms differentially regulate brain amyloid-β peptide clearance. Sci Transl Med.

[13] Dikranian K (2012). Ultrastructural studies in APP/PS1 mice expressing human ApoE isoforms: implications for Alzheimer’s disease. Int J Clin Exp Pathol.

[14] Shi Y (2017). ApoE4 markedly exacerbates tau-mediated neurodegeneration in a mouse model of tauopathy. Nature.

[15] Kadotani H (2001). Association between apolipoprotein E epsilon4 and sleep-disordered breathing in adults. JAMA.

[16] Yaffe K (2011). Sleep-disordered breathing, hypoxia, and risk of mild cognitive impairment and dementia in older women. JAMA.

[17] Gottlieb DJ (2004). APOE epsilon4 is associated with obstructive sleep apnea/hypopnea: the Sleep Heart Health Study. Neurology.

[18] Young T (1993). The occurrence of sleep-disordered breathing among middle-aged adults. N Engl J Med.

[19] Bixler EO (1998). Effects of age on sleep apnea in men: I. Prevalence and severity. Am J Respir Crit Care Med.

[20] Koo KYG (2019). Abnormal sleep behaviours across the spectrum of Alzheimer’s disease severity: influence of APOE genotypes and lewy bodies. Curr Alzheimer Res.

[21] Wang C, Holtzman DM (2020). Bidirectional relationship between sleep and Alzheimer’s disease: role of amyloid, tau, and other factors. Neuropsychopharmacology.

[22] Fagan AM (2002). Human and murine ApoE markedly alters A beta metabolism before and after plaque formation in a mouse model of Alzheimer’s disease. Neurobiol Dis.

[23] Huynh TV (2019). Lack of hepatic apoE does not influence early Aβ deposition: observations from a new APOE knock-in model. Mol Neurodegener.

[24] Bellesi M (2017). Sleep loss promotes astrocytic phagocytosis and microglial activation in mouse cerebral cortex. J Neurosci.

[25] Leyns CEG (2019). TREM2 function impedes tau seeding in neuritic plaques. Nat Neurosci.

[26] He Z (2018). Amyloid-β plaques enhance Alzheimer’s brain tau-seeded pathologies by facilitating neuritic plaque tau aggregation. Nat Med.

[27] Jain N (2022). Chronic TREM2 activation exacerbates Aβ-associated tau seeding and spreading. J Exp Med.

[28] Xie L (2013). Sleep drives metabolite clearance from the adult brain. Science.

[29] Iliff JJ (2012). A paravascular pathway facilitates CSF flow through the brain parenchyma and the clearance of interstitial solutes, including amyloid β. Sci Transl Med.

[30] Xu Z (2015). Deletion of aquaporin-4 in APP/PS1 mice exacerbates brain Aβ accumulation and memory deficits. Mol Neurodegener.

[31] Ishida K (2022). Glymphatic system clears extracellular tau and protects from tau aggregation and neurodegeneration. J Exp Med.

[32] Matthes F (2021). An improved method for physical separation of cerebral vasculature and parenchyma enables detection of blood-brain-barrier dysfunction. NeuroSci.

[33] Roh JH (2012). Disruption of the sleep-wake cycle and diurnal fluctuation of β-amyloid in mice with Alzheimer’s disease pathology. Sci Transl Med.

[34] Nebes RD (2009). Self-reported sleep quality predicts poor cognitive performance in healthy older adults. J Gerontol B Psychol Sci Soc Sci.

[35] Xu L (2011). Short or long sleep duration is associated with memory impairment in older Chinese: the Guangzhou Biobank Cohort Study. Sleep.

[36] Wennberg AMV (2017). Sleep disturbance, cognitive decline, and dementia: a review. Semin Neurol.

[37] Gent TC (2018). Sleep-wake control and the thalamus. Curr Opin Neurobiol.

[38] Victor MB (2022). Lipid accumulation induced by APOE4 impairs microglial surveillance of neuronal-network activity. Cell Stem Cell.

[39] Yuan P (2016). TREM2 haplodeficiency in mice and humans impairs the microglia barrier function leading to decreased amyloid compaction and severe axonal dystrophy. Neuron.

[40] Wang Y (2016). TREM2-mediated early microglial response limits diffusion and toxicity of amyloid plaques. J Exp Med.

[41] Condello C (2015). Microglia constitute a barrier that prevents neurotoxic protofibrillar Aβ42 hotspots around plaques. Nat Commun.

[42] Hurtado DE (2010). Abeta accelerates the spatiotemporal progression of tau pathology and augments tau amyloidosis in an Alzheimer mouse model. Am J Pathol.

[43] Bennett RE (2017). Enhanced Tau aggregation in the presence of amyloid β. Am J Pathol.

[44] Gotz J (2001). Formation of neurofibrillary tangles in P301l tau transgenic mice induced by Abeta 42 fibrils. Science.

[45] Gratuze M (2021). Activated microglia mitigate Aβ-associated tau seeding and spreading. J Exp Med.

[46] Ulland TK (2017). TREM2 maintains microglial metabolic fitness in Alzheimer’s Disease. Cell.

[48] Liu CC (2017). ApoE4 accelerates early seeding of amyloid pathology. Neuron.

[49] Simon M (2022). Loss of perivascular aquaporin-4 localization impairs glymphatic exchange and promotes amyloid β plaque formation in mice. Alzheimers Res Ther.

[50] Drieu A (2022). Parenchymal border macrophages regulate the flow dynamics of the cerebrospinal fluid. Nature.

[51] Holth JK (2017). Altered sleep and EEG power in the P301S Tau transgenic mouse model. Ann Clin Transl Neurol.

[52] Gratuze M (2022). APOE antibody inhibits aβ-associated tau seeding and spreading in a mouse model. Ann Neurol.

[53] Carrier J (2017). Sex differences in age-related changes in the sleep-wake cycle. Front Neuroendocrinol.

[54] Paul KN (2006). Diurnal sex differences in the sleep-wake cycle of mice are dependent on gonadal function. Sleep.

[55] Franken P (2006). NPAS2 as a transcriptional regulator of non-rapid eye movement sleep: genotype and sex interactions. Proc Natl Acad Sci U S A.

[56] Koehl M (2006). Sex differences in sleep: the response to sleep deprivation and restraint stress in mice. Sleep.

[57] Guo JL (2016). Unique pathological tau conformers from Alzheimer’s brains transmit tau pathology in nontransgenic mice. J Exp Med.

[58] Donohue KD (2008). Assessment of a non-invasive high-throughput classifier for behaviours associated with sleep and wake in mice. Biomed Eng Online.

[59] Mang GM (2014). Evaluation of a piezoelectric system as an alternative to electroencephalogram/ electromyogram recordings in mouse sleep studies. Sleep.

[60] Boulay AC Purification of mouse brain vessels. J Vis Exp.

