# Supplementary material for: APOE-ε4 synergizes with sleep disruption to accelerate Aβ deposition and Aβ-associated tau seeding and spreading
Source: J Clin Invest. 2023 Jul 17;133(14):e169131. doi: 10.1172/JCI169131 (PMC10351966; doi:10.1172/JCI169131)
Supplement: Supplemental data [file jci-133-169131-s010.pdf]

Title: *APOE-ε4* synergizes with sleep disruption to accelerate Aβ deposition and Aβ-associated tau seeding and spreading

Authors and affiliations: Chanung Wang[1], Aishwarya Nambiar[1], Michael R. Strickland[1], Choonghee Lee[1], Samira Parhizkar[1], Alec C. Moore[1], Erik S. Musiek[1], Jason D. Ulrich[1], and David M. Holtzman[1*]

[1]Department of Neurology, Hope Center for Neurological Disorders, Knight Alzheimer's Disease Research Center, Washington University School of Medicine, St. Louis, MO USA

Corresponding Author: [*]For correspondence, contact David M. Holtzman, Department of Neurology, Hope Center for Neurological Disorders, Knight Alzheimer's Disease Research Center, Washington University School of Medicine, 660 S. Euclid Avenue, Box 8111, St. Louis, Missouri, 63110, USA.; Email: holtzman@wustl.edu.

Conflict of interest statement: D.M.H. co-founded, has equity, and is on the scientific advisory board of C2N Diagnostics. D.M.H. is on the scientific advisory board of Denali, Cajal Neuroscience, and Genentech and consults for Alector. All other authors have no competing interests.

Supplementary figures and figure legends

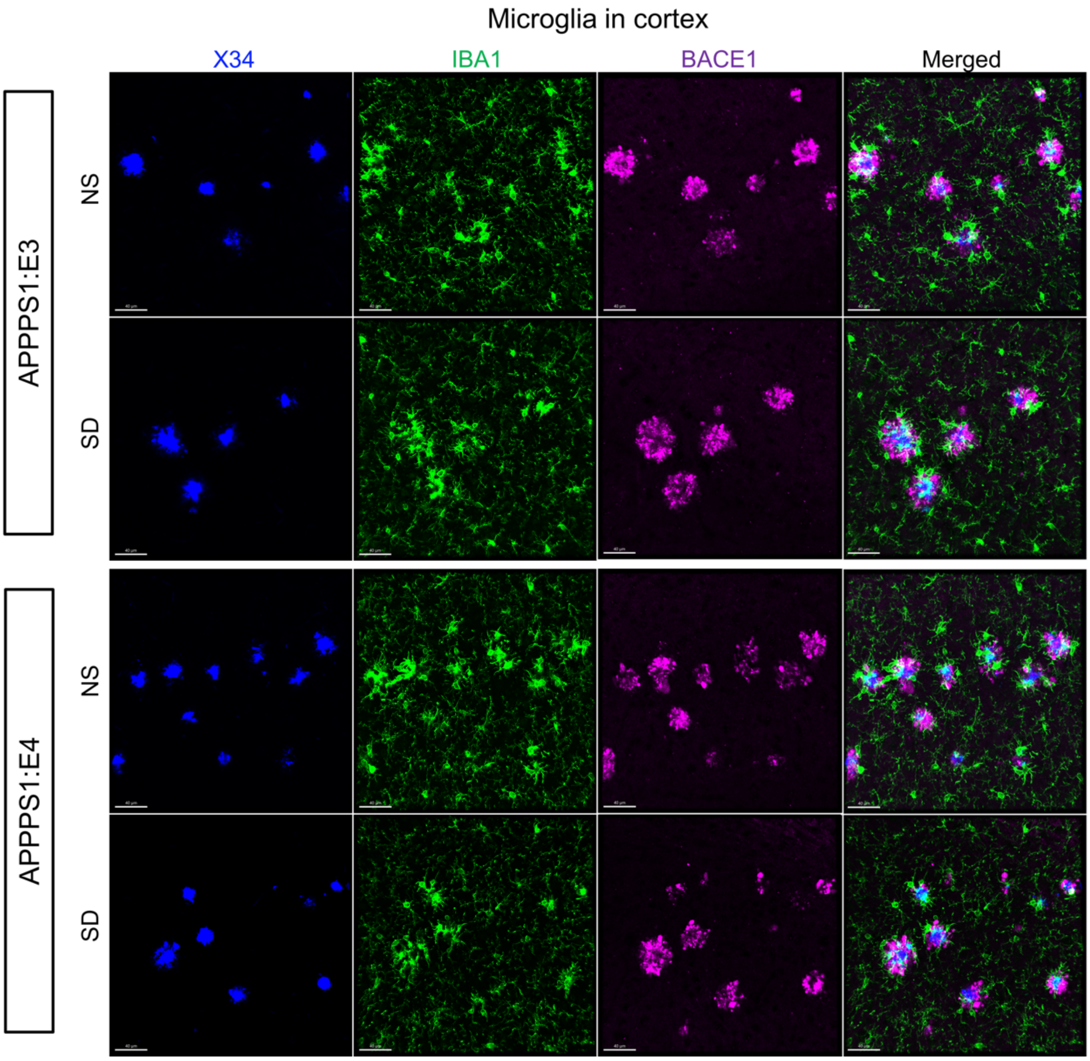

Supplementary figure 1. Sleep deprivation in APPPS1 mice differently affects microglia clustering and dystrophic neurite formation around plaques in an apoE-isoform dependent manner. Confocal images of IBA1-labeled microglia (green) and neuritic dystrophy (BACE1, magenta co-stained around X34+ plaques (blue) in cortex of APPPS1:E3 and APPPS1:E4 mice. Scale bar, 40 µm.

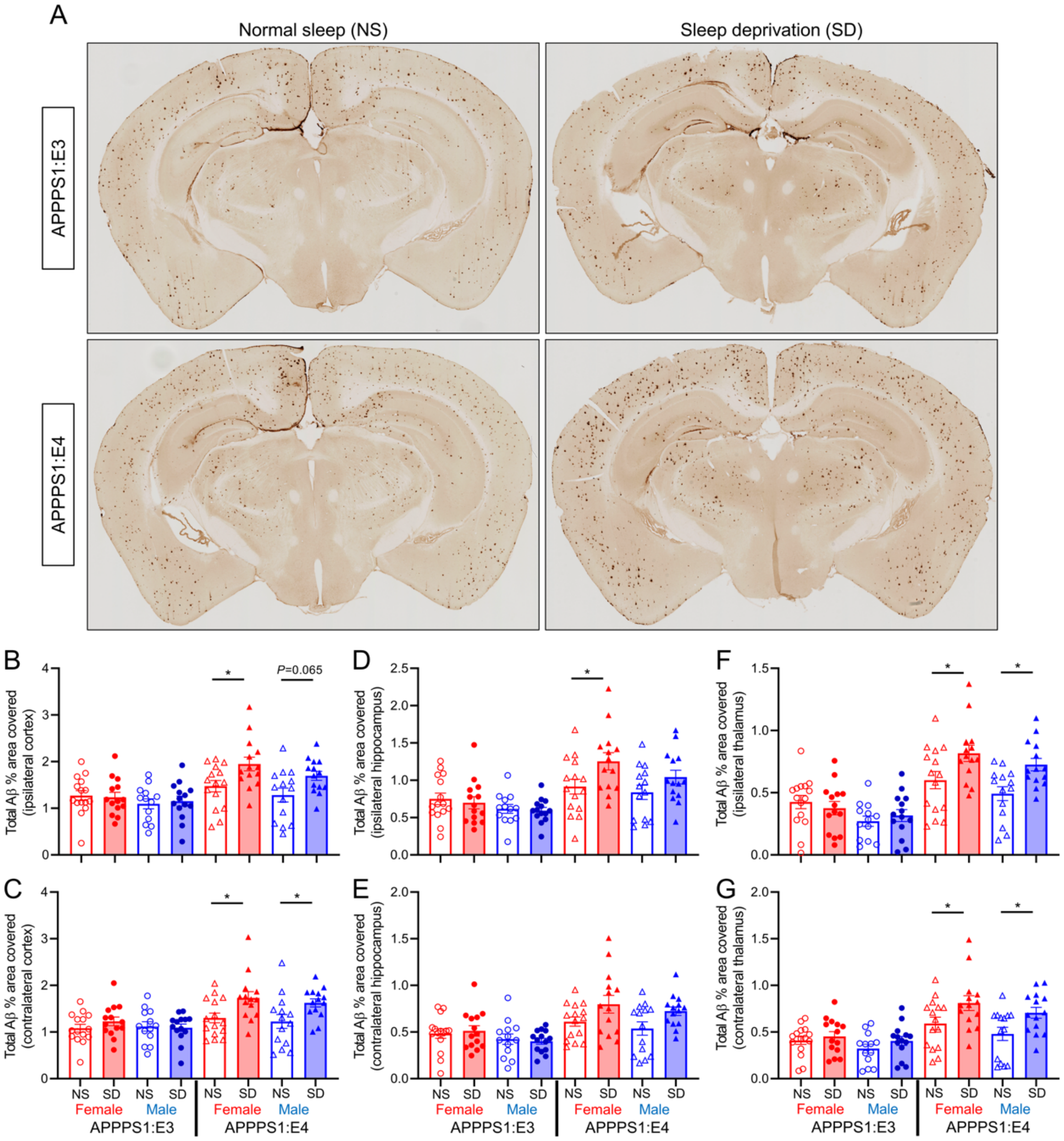

Supplementary figure 2. Sleep deprivation with AD tau seeding in APPPS1 mice significantly increases amyloid plaque deposition in the presence of APOE4 but not APOE3. **(A)** Representative images of brain sections stained with anti-Aβ antibody (HJ3.4-biotin) in AD-tau-injected APPPS1:E3 and APPPS1:E4 mice from normal sleep (NS) or sleep deprivation (SD) groups. Scale bar, 500 µm. **(B-G)** Quantification of percentage area covered by Aβ+ staining in the ipsi- and contralateral cortices (**B** and **C**, respectively), hippocampi (**D** and **E**, respectively), and thalami (**F** and **G**, respectively) and of AD-tau-injected APPPS1:E3 and APPPS1:E4 mice (n = 13-15 per group). Data are presented as mean ± S.E.M. Significance was determined by three-way ANOVA- Sidak's multiple comparison test (sex, apoE genotype, and sleep condition). * $P<0.05$, ** $P<0.01$.

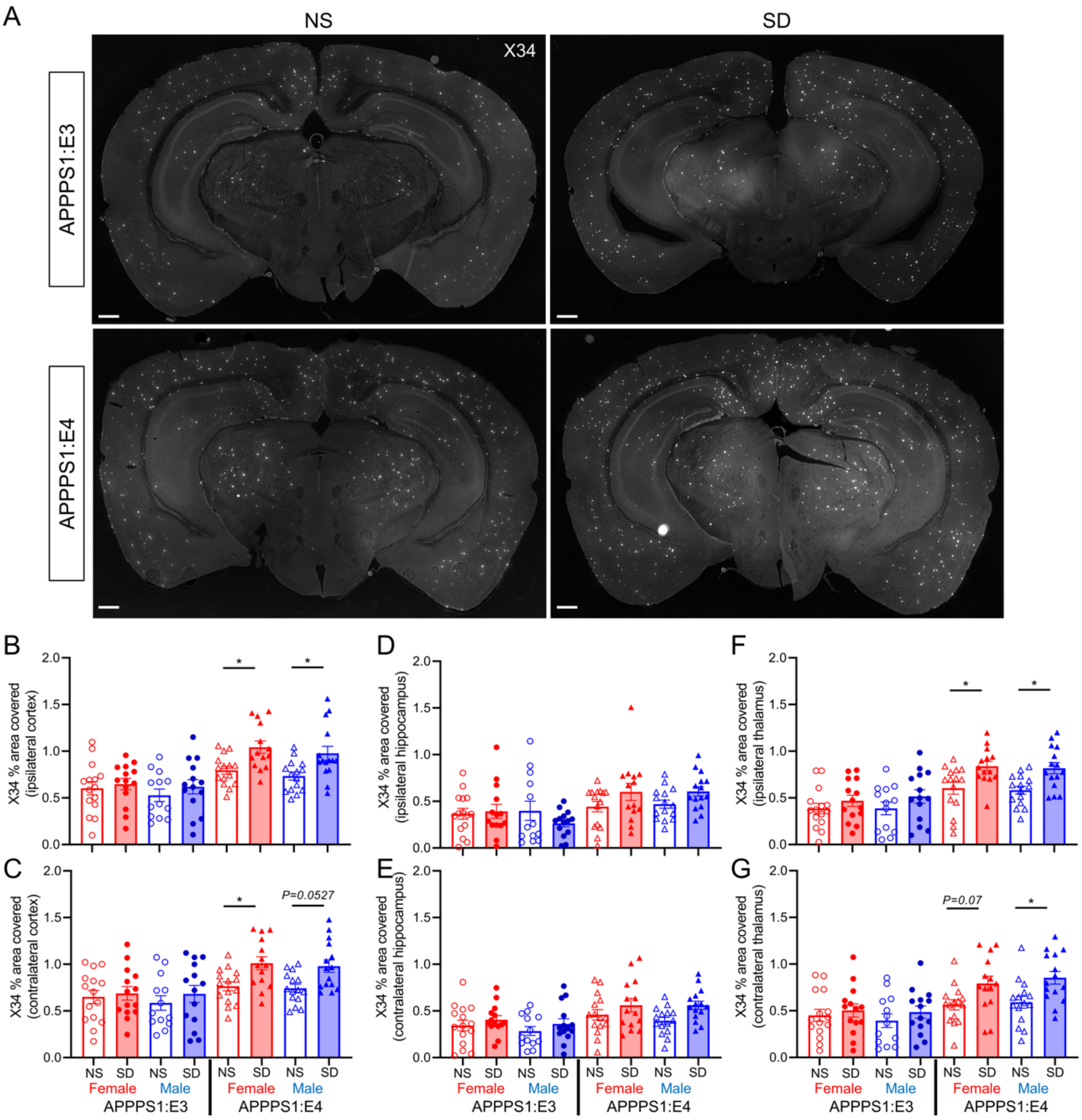

Supplementary figure 3. Sleep deprivation with AD tau seeding in APPPS1 mice increases fibrillar plaques in an apoE-isoform dependent manner. (A) Representative images of brain sections stained with X34 in AD-tau-injected APPPS1:E3 and E4 mice from normal sleep (NS) or sleep deprivation (SD) groups. Scale bar, 500 µm. **(B-G)** Quantification of percentage area covered by X-34 staining in the ipsi- and contralateral cortices (**B** and **C**, respectively), hippocampi (**D** and **E**, respectively), and thalami (**F** and **G**, respectively) of AD-tau-injected APPPS1:E3 and APPPS1:E4 mice. Data are presented as mean ± S.E.M. Significance was determined by three-way ANOVA- Sidak's multiple comparison test (sex, apoE genotype, and sleep condition). * P<0.05, ** P<0.01.

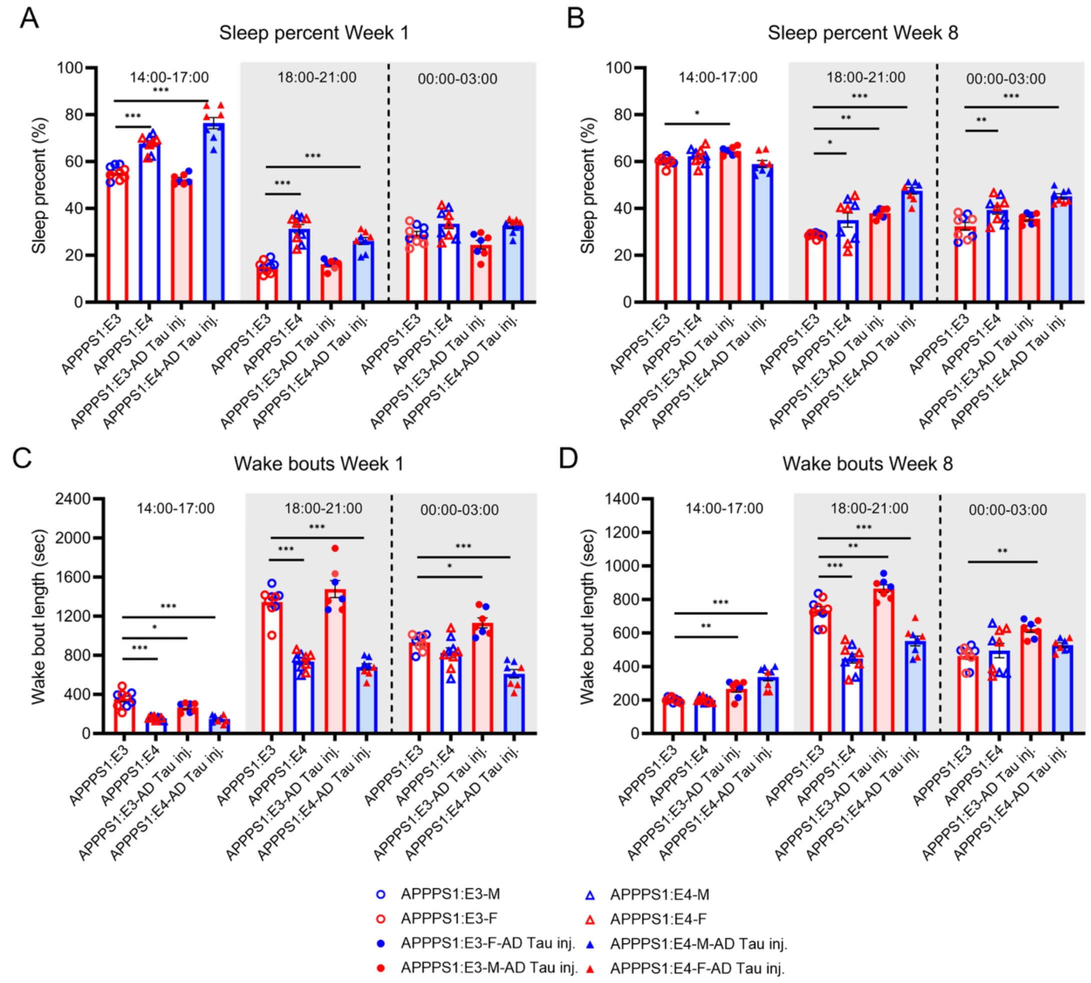

Supplementary figure 4. Aβ deposition and peri-plaque NP-tau pathology significantly affects sleep rebound behaviors in the presence of APOE4 but not APOE3. (A-D) Sleep-wake recording data were analyzed with three-time points (14:00-17:00, 18:00-21:00, and 00:00-03:00) to investigate sleep rebound behaviors of APPPS1:E3 and E4 mice after SD. Average sleep times as percentages and average wake bout length of each group for 14:00-17:00, 18:00-21:00, and 00:00-03:00 for the first week of SD (**A and C** respectively) and the eight week of SD (**B and D** respectively). There was a significant effect of apoE genotype and AD-tau injection in the **14:00-17:00** (**A**: apoE genotype $F(1,25) = 257.0$, $p < 0.0001$; AD tau injection $F(1,25) = 6.631$, $p = 0.0163$; Sex $F(1,25) = 2.343$, $p = 0.1384$; apoE genotype X AD tau injection $F(1,25) = 26.53$, $p < 0.0001$, **B**: apoE genotype $F(1,25) = 3.168$, $p = 0.0872$; AD tau injection $F(1,25) = 0.2392$, $p = 0.6290$; Sex $F(1,25) = 1.589$, $p = 0.2191$; apoE genotype X AD tau injection $F(1,25) = 16.27$, $p = 0.0005$, **C**: apoE genotype $F(1,25) = 61.97$, $p < 0.0001$; AD tau injection $F(1,25) = 5.323$, $p = 0.0296$; Sex $F(1,25) = 0.1706$, $p = 0.6831$; apoE genotype X AD tau injection $F(1,25) = 3.611$, $p = 0.0690$, **D**: apoE genotype $F(1,25) = 9.021$, $p = 0.0060$; AD tau injection $F(1,25) = 72.59$, $p < 0.0001$; Sex $F(1,25) = 4.246$, $p = 0.0499$; apoE genotype X AD tau injection $F(1,25) = 9.021$, $p = 0.0060$), **18:00-21:00** (**A**: apoE genotype $F(1,25) = 89.76$, $p < 0.0001$; AD tau injection $F(1,25) = 2.240$, $p = 0.1470$; Sex $F(1,25) = 0.004377$, $p = 0.9478$; apoE genotype X AD tau injection $F(1,25) = 5.497$, $p = 0.0273$, **B**: apoE genotype $F(1,25) = 17.90$, $p = 0.0003$; AD tau injection $F(1,25) = 33.02$, $p < 0.0001$; Sex $F(1,25) = 1.077$, $p = 0.3094$; apoE genotype X AD tau injection $F(1,25) = 0.6808$, $p = 0.4171$, **C**: apoE genotype $F(1,25) = 194.7$, $p < 0.0001$; AD tau injection $F(1,25) = 0.3860$, $p = 0.5400$; Sex $F(1,25) = 0.1294$, $p = 0.7221$; apoE genotype X AD tau injection $F(1,25) = 2.844$, $p =$

0.1042, **D**: apoE genotype $F(1,25) = 123.3$, $p < 0.0001$; AD tau injection $F(1,25) = 20.03$, $p = 0.0001$; Sex $F(1,25) = 0.01169$, $p = 0.9148$; apoE genotype X AD tau injection $F(1,25) = 0.3362$, $p = 0.5672$), , and **00:00-03:00** (**A**: apoE genotype $F(1,25) = 13.59$, $p = 0.0011$; AD tau injection $F(1,25) = 2.315$, $p = 0.1406$; Sex $F(1,25) = 0.01181$, $p = 0.9143$; apoE genotype X AD tau injection $F(1,25) = 1.099$, $p = 0.3045$, **B**: apoE genotype $F(1,25) = 29.27$, $p < 0.0001$; AD tau injection $F(1,25) = 9.051$, $p = 0.0059$; Sex $F(1,25) = 0.6536$, $p = 0.4264$; apoE genotype X AD tau injection $F(1,25) = 0.6618$, $p = 0.4236$, **C**: apoE genotype $F(1,25) = 49.67$, $p < 0.0001$; AD tau injection $F(1,25) = 0.04070$, $p = 0.8417$; Sex $F(1,25) = 0.4226$, $p = 0.5216$; apoE genotype X AD tau injection $F(1,25) = 21.17$, $p < 0.0001$, **D**: apoE genotype $F(1,25) = 1.153$, $p = 0.2931$; AD tau injection $F(1,25) = 10.26$, $p = 0.0037$; Sex $F(1,25) = 0.01947$, $p = 0.8901$; apoE genotype X AD tau injection $F(1,25) = 4.288$, $p = 0.0488$). In (A-D), APPPS1:E3, APPPS1:E3-AD Tau inj., and APPPS1:E4-AD Tau inj. : n=8 (4 males and 4 females); APPPS1:E4: n=9 (4 males and 5 females). Data are presented as mean ± S.E.M. Significance was determined by three-way ANOVA followed by a Tukey's post hoc test (apoE genotype, AD tau injection, and sex). * P<0.05, ** P<0.01, *** P<0.001. F* or M* indicates significance in females or males respectively.

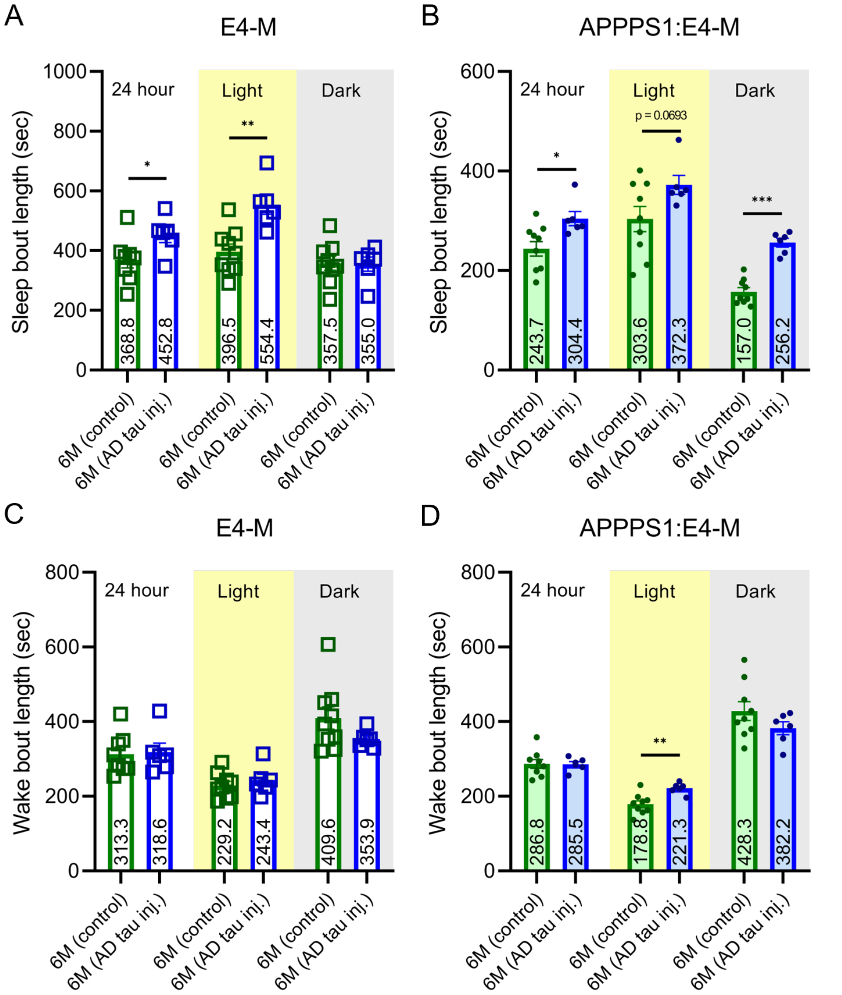

Supplementary figure 5. Aβ deposition and peri-plaque NP-tau pathology significantly affects sleep behaviors in the presence of APOE4 but not APOE3.
(A-D) Average sleep bout length and average wake bout length of each group for 24 hours, light phase, and dark phase in E4 male mice **(A and C** respectively**)** and APPPS1:E4 male mice (**B and D** respectively). In A-D, there were significant effects of AD-tau injection (A) Sleep bout length in E4 male mice, 24 hour ($t = 2.224$, $p = 0.0461$); Light ($t = 3.905$, $p = 0.0018$), (B) Sleep bout length in APPPS1:E4 male mice 24 hour ($t = 2.804$, $p = 0.0141$); Light ($t = 1.980$, $p = 0.0693$); Dark ($t = 7.818$, $p < 0.0001$, **(C)** Wake bout length in E4 male mice, no significance, **(D)** Wake bout length in APPPS1:E4 male mice, Light ($t = 3.499$, $p = 0.0039$). 6M (control): n=9; 6M (AD tau inj.): n=6. Data are presented as mean ± S.E.M. Significance was determined by unpaired Student’s t-test (6M (control) vs. 6M (AD tau inj.)). * *P*<0.05, ** *P*<0.01, *** *P*<0.001.

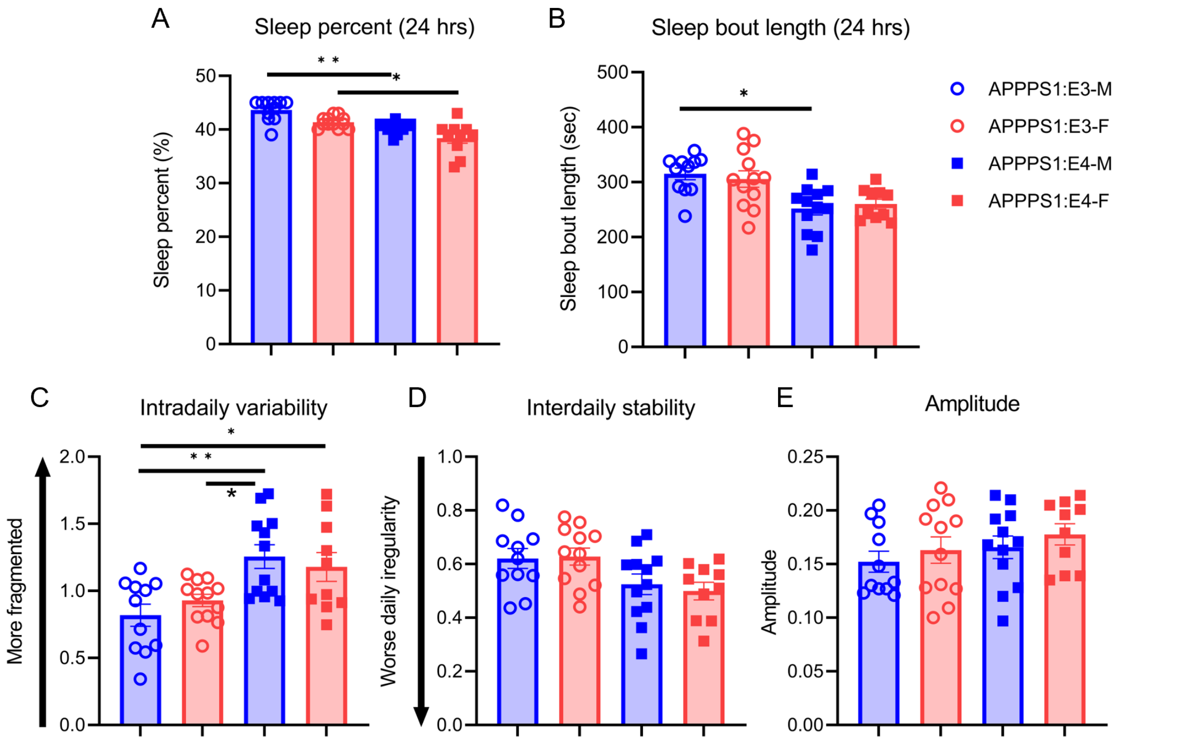

Supplementary figure 6. *APOE4* genotype modifies the various parameters of sleep-wake regularity. (A and B) Average sleep percent and sleep bout length of each group for 24 hours in APPPS1:E3 and APPPS1:E4 mice, respectively. Mean Sleep percent (**A**: apoE genotype $F(1,41) = 32.03$, $p < 0.0001$; Sex $F(1,41) = 12.94$, $p = 0.0009$, Average sleep bout length (**B**: apoE genotype $F(1,41) = 20.17$, $p < 0.0001$; Sex $F(1,41) = 0.002339$, $p = 0.9617$). **(C)** Intradaily Variability (IV) of each group (apoE genotype $F(1,41) = 17.43$, $p = 0.0002$; Sex $F(1,41) = 0.0389$, $p = 0.8446$), **(D)** Interdaily Stability (IS) of each group (apoE genotype $F(1,41) = 10.10$, $p = 0.0028$; Sex $F(1,41) = 0.06409$, $p = 0.8014$), **(E)** amplitude of activity of each group (apoE genotype $F(1,41) = 1.702$, $p = 0.1994$; Sex $F(1,41) = 1.120$, $p = 0.2961$): n=10-12. Data are presented as mean ± SEM. Significance was determined by Two-way ANOVA followed by a Sidak's multiple comparison test (apoE genotype and sex). * $P<0.05$, ** $P<0.01$.

Table S1. Detailed statistical information		
**Figure**	**Sample size**	**Statistic information table**
Figure 1D	APPPS1:E3-F-con: n=15; APPPS1:E3-F-SD: n=13; APPPS1:E3-M-con: n=14; APPPS1:E3-M-SD: n=13; APPPS1:E4-F-con: n=15; APPPS1:E4-F-SD: n=13; APPPS1:E4-M-con: n=14; APPPS1:E4-M-SD: n=13;	Three-way ANOVA, apoE genotype F(1,103) = 32.79, p < 0.0001; Sleep condition F(1,103) =26.45, p < 0.0001; apoE genotype X Sleep condition F(1,103) =7.897, p = 0.0059 in AP-PPS1:E3-con vs. APPPS1:E3-SD and AP-PPS1:E4-con vs. APPPS1:E4-SD
Figure 1E		Three-way ANOVA, apoE genotype (F(1,103) = 62.73, p < 0.001; Sleep condition (F(1,103) =30.27, p < 0.0001; apoE genotype X Sleep condition F(1,103) =4.157, p = 0.044 in AP-PPS1:E3-con vs. APPPS1:E3-SD and AP-PPS1:E4-con vs. APPPS1:E4-SD
Figure 1E		Three-way ANOVA, apoE genotype (F(1,103) = 62.73, p < 0.001; Sleep condition (F(1,103) =30.27, p < 0.0001; apoE genotype X Sleep condition F(1,103) =15.31, p < 0.001 in AP-PPS1:E3-con vs. APPPS1:E3-SD and AP-PPS1:E4-con vs. APPPS1:E4-SD
Figure 2B	APPPS1:E3-F-con: n=15; APPPS1:E3-F-SD: n=12; APPPS1:E3-M-con: n=13; APPPS1:E3-M-SD: n=14; APPPS1:E4-F-con: n=15; APPPS1:E4-F-SD: n=13; APPPS1:E4-M-con: n=15; APPPS1:E4-M-SD: n=14;	Three-way ANOVA, apoE genotype F(1,101) = 19.46, p < 0.0001; Sleep condition F(1,101) =22.52, p < 0.0001; apoE genotype X Sleep condition F(1,101) =4.770, p = 0.0313 in AP-PPS1:E3-con vs. APPPS1:E3-SD and AP-PPS1:E4-con vs. APPPS1:E4-SD
Figure 2C		Three-way ANOVA, apoE genotype F(1,101) = 16.06, p < 0.0001; Sleep condition F(1,101) =17.53, p < 0.0001; apoE genotype X Sleep condition F(1,101) =5.533, p = 0.0206 in AP-PPS1:E3-con vs. APPPS1:E3-SD and AP-PPS1:E4-con vs. APPPS1:E4-SD
Figure 2D		Three-way ANOVA, apoE genotype F(1,101) = 28.39, p < 0.0001; Sleep condition F(1,101) =20.67, p < 0.0001; APOE istofom X Sleep condition F(1,101) =10.90, p = 0.0013 in AP-PPS1:E3-con vs. APPPS1:E3-SD and AP-PPS1:E4-con vs. APPPS1:E4-SD
Figure 3C	APPPS1:E3-F-con: n=14; APPPS1:E3-F-SD: n=12; APPPS1:E3-M-con: n=12; APPPS1:E3-M-SD: n=14; APPPS1:E4-F-con: n=14; APPPS1:E4-F-SD: n=13; APPPS1:E4-M-con: n=15; APPPS1:E4-M-SD: n=14;	Three-way ANOVA, apoE genotype F(1,99) = 0.5864, p = 0.5864; Sleep condition F(1,99) =15.26, p = 0.0002 in APPPS1:E3-con vs. APPPS1:E3-SD and APPPS1:E4-con vs. AP-PPS1:E4-SD
Figure 3D		No significance, Three-way ANOVA, apoE genotype F(1,99) = 0.0423, p = 0.9948; Sleep condition F(1,99) =2.122, p = 0.1484 in AP-PPS1:E3-con vs. APPPS1:E3-SD and AP-PPS1:E4-con vs. APPPS1:E4-SD

Figure 3E		Three-way ANOVA, apoE genotype F(1,99) = 14.91, p = 0.0002; Sleep condition F(1,99) =10.889, p = 0.0013; apoE genotype X Sleep condition F(1,99) =10.05, p = 0.0020 in APPPS1:E3-con vs. APPPS1:E3-SD and APPPS1:E4-con vs. APPPS1:E4-SD
Figure 3F	APPPS1:E3-F-con: n=14; APPPS1:E3-F-SD: n=13; APPPS1:E3-M-con: n=13; APPPS1:E3-M-SD: n=14; APPPS1:E4-F-con: n=14; APPPS1:E4-F-SD: n=13; APPPS1:E4-M-con: n=14; APPPS1:E4-M-SD: n=15;	Three-way ANOVA, apoE genotype F(1,103) = 0.1859, p = 0.6673; Sleep condition F(1,103) =14.63, p = 0.0002; apoE genotype X Sleep condition F(1,103) =1.721, p = 0.1924 in APPPS1:E3-con vs. APPPS1:E3-SD and APPPS1:E4-con vs. APPPS1:E4-SD
Figure 3G		Three-way ANOVA, apoE genotype F(1,103) = 0.00046, p = 0.9829; Sleep condition F(1,103) =15.88, p = 0.0001; apoE genotype X Sleep condition F(1,103) =2.954, p = 0.0887 in APPPS1:E3-con vs. APPPS1:E3-SD and APPPS1:E4-con vs. APPPS1:E4-SD
Figure 3H		Three-way ANOVA, apoE genotype F(1,103) = 0.02592, p = 0.8724; Sleep condition F(1,103) =21.21, p < 0.0001; apoE genotype X Sleep condition F(1,103) =1.035, p = 0.3113 in APPPS1:E3-con vs. APPPS1:E3-SD and APPPS1:E4-con vs. APPPS1:E4-SD
Figure 3I	APPPS1:E3-F-con: n=14; APPPS1:E3-F-SD: n=12; APPPS1:E3-M-con: n=12; APPPS1:E3-M-SD: n=14; APPPS1:E4-F-con: n=14; APPPS1:E4-F-SD: n=13; APPPS1:E4-M-con: n=15; APPPS1:E4-M-SD: n=14;	Three-way ANOVA, apoE genotype F(1,99) = 1.434, p = 0.234; Sleep condition F(1,99) =4.910, p = 0.029; apoE genotype X Sleep condition F(1,99) =0.1309, p = 0.718 in APPPS1:E3-con vs. APPPS1:E3-SD and APPPS1:E4-con vs. APPPS1:E4-SD
Figure 3J		Three-way ANOVA, apoE genotype F(1,99) = 0.5423, p = 0.463; Sleep condition F(1,99) =10.26, p = 0.002; apoE genotype X Sleep condition F(1,99) =3.587, p = 0.061 in APPPS1:E3-con vs. APPPS1:E3-SD and APPPS1:E4-con vs. APPPS1:E4-SD
Figure 3K		Three-way ANOVA, apoE genotype F(1,99) = 0.4540, p = 0.502; Sleep condition F(1,99) =9.131, p = 0.003; apoE genotype X Sleep condition F(1,99) =2.880, p = 0.093 in APPPS1:E3-con vs. APPPS1:E3-SD and APPPS1:E4-con vs. APPPS1:E4-SD
Figure 4E	APPPS1:E3-F-con: n=15; APPPS1:E3-F-SD: n=14; APPPS1:E3-M-con: n=13; APPPS1:E3-M-SD: n=13; APPPS1:E4-F-con: n=15; APPPS1:E4-F-SD: n=14; APPPS1:E4-M-con: n=15; APPPS1:E4-M-SD: n=15;	Three-way ANOVA, apoE genotype F(1,106) = 23.09, p < 0.0001; Sleep condition F(1,106) =15.65, p < 0.0001; apoE genotype X Sleep condition F(1,106) =15.74, p = 0.001 in APPPS1:E3-con vs. APPPS1:E3-SD and APPPS1:E4-con vs. APPPS1:E4-SD
Figure 4F		Three-way ANOVA, apoE genotype F(1,106) = 9.181, p = 0.0031; Sleep condition F(1,106) =6.651, p = 0.0113; apoE genotype X Sleep

		condition F(1,106) =15.57, p = 0.001 in AP-PPS1:E3-con vs. APPPS1:E3-SD and AP-PPS1:E4-con vs. APPPS1:E4-SD
Figure 4G		Three-way ANOVA, apoE genotype F(1,106) = 8.321, p = 0.005; Sleep condition F(1,106) =3.060, p = 0.083; apoE genotype X Sleep condition F(1,106) =1.894, p = 0.172 in AP-PPS1:E3-con vs. APPPS1:E3-SD and AP-PPS1:E4-con vs. APPPS1:E4-SD
Figure 4H		No significance, Three-way ANOVA, apoE genotype F(1,106) = 0.5464, p = 0.461; Sleep condition F(1,106) =0.1494, p = 0.700; APOEi-soformm X Sleep condition F(1,106) =3.111, p = 0.081 in APPPS1:E3-con vs. APPPS1:E3-SD and APPPS1:E4-con vs. APPPS1:E4-SD
Figure 5C	APPPS1:E3-F-con: n=14; APPPS1:E3-F-SD: n=14; APPPS1:E3-M-con: n=13; APPPS1:E3-M-SD: n=14; APPPS1:E4-F-con: n=15; APPPS1:E4-F-SD: n=14; APPPS1:E4-M-con: n=15; APPPS1:E4-M-SD: n=15;	Three-way ANOVA, apoE genotype F(1,106) = 34.33, p < 0.001; Sleep condition F(1,106) =15.11, p < 0.001; apoE genotype X Sleep condition F(1,106) =3.524, p = 0.074 in AP-PPS1:E3-con vs. APPPS1:E3-SD and AP-PPS1:E4-con vs. APPPS1:E4-SD
Figure 5D		Three-way ANOVA, apoE genotype F(1,106) = 9.902, p = 0.0021; Sleep condition F(1,106) =11.20, p = 0.0011; apoE genotype X Sleep condition F(1,106) =4.832, p = 0.0301 in AP-PPS1:E3-con vs. APPPS1:E3-SD and AP-PPS1:E4-con vs. APPPS1:E4-SD
Figure 5E		Three-way ANOVA, apoE genotype F(1,106) = 18.40, p < 0.001; Sleep condition F(1,106) =0.02165, p = 0.883; apoE genotype X Sleep condition F(1,106) =0.6513, p = 0.421 in AP-PPS1:E3-con vs. APPPS1:E3-SD and AP-PPS1:E4-con vs. APPPS1:E4-SD
Figure 5F		Three-way ANOVA, apoE genotype F(1,106) = 13.00, p = 0.0005; Sleep condition F(1,106) =8.949, p = 0.0035; apoE genotype X Sleep condition F(1,106) =5.618, p = 0.0196 in AP-PPS1:E3-con vs. APPPS1:E3-SD and AP-PPS1:E4-con vs. APPPS1:E4-SD
Figure 5G		Three-way ANOVA, apoE genotype F(1,106) = 30.54, p < 0.001; Sleep condition F(1,106) =8.163, p = 0.005; apoE genotype X Sleep condition F(1,106) =3.452, p = 0.066 in AP-PPS1:E3-con vs. APPPS1:E3-SD and AP-PPS1:E4-con vs. APPPS1:E4-SD
Figure 5H		Three-way ANOVA, apoE genotype F(1,106) = 9.148, p = 0.0031; Sleep condition F(1,106) =1.558, p = 0.2147; apoE genotype X Sleep condition F(1,106) =1.944, p = 0.1661 in AP-PPS1:E3-con vs. APPPS1:E3-SD and AP-PPS1:E4-con vs. APPPS1:E4-SD

Figure 6C	APPPS1:E3-F-con: n=14;<br>APPPS1:E3-F-SD: n=14;<br>APPPS1:E3-M-con: n=13;<br>APPPS1:E3-M-SD: n=14;<br>APPPS1:E4-F-con: n=15;<br>APPPS1:E4-F-SD: n=14;<br>APPPS1:E4-M-con: n=15;<br>APPPS1:E4-M-SD: n=15;	Three-way ANOVA, apoE genotype $F(1,106) = 0.1319$, $p = 0.7172$; Sleep condition $F(1,106) = 61.00$, $p < 0.0001$; apoE genotype X Sleep condition $F(1,106) = 14.89$, $p = 0.0002$ in APPPS1:E3-con vs. APPPS1:E3-SD and APPPS1:E4-con vs. APPPS1:E4-SD
Figure 6D		Three-way ANOVA, apoE genotype $F(1,106) = 8.361$, $p = 0.0047$; Sleep condition $F(1,106) = 41.63$, $p < 0.0001$; apoE genotype X Sleep condition $F(1,106) = 7.536$, $p = 0.0071$ in APPPS1:E3-con vs. APPPS1:E3-SD and APPPS1:E4-con vs. APPPS1:E4-SD
Figure 6E		Three-way ANOVA, apoE genotype $F(1,106) = 0.04477$, $p = 0.8328$; Sleep condition $F(1,106) = 15.81$, $p = 0.0001$; apoE genotype X Sleep condition $F(1,106) = 2.830$, $p = 0.0955$ in APPPS1:E3-con vs. APPPS1:E3-SD and APPPS1:E4-con vs. APPPS1:E4-SD
Figure 6F		Three-way ANOVA, apoE genotype $F(1,106) = 0.0032$, $p = 0.9543$; Sleep condition $F(1,106) = 44.51$, $p < 0.0001$; apoE genotype X Sleep condition $F(1,106) = 7.305$, $p = 0.0080$ in APPPS1:E3-con vs. APPPS1:E3-SD and APPPS1:E4-con vs. APPPS1:E4-SD
Figure 6G		Three-way ANOVA, apoE genotype $F(1,106) = 3.71$, $p = 0.0568$; Sleep condition $F(1,106) = 25.85$, $p < 0.0001$; apoE genotype X Sleep condition $F(1,106) = 4.543$, $p = 0.0354$ in APPPS1:E3-con vs. APPPS1:E3-SD and APPPS1:E4-con vs. APPPS1:E4-SD
Figure 6H		Three-way ANOVA, apoE genotype $F(1,106) = 8.197$, $p = 0.0051$; Sleep condition $F(1,106) = 18.37$, $p < 0.001$; apoE genotype X Sleep condition $F(1,106) = 2.120$, $p = 0.1483$ in APPPS1:E3-con vs. APPPS1:E3-SD and APPPS1:E4-con vs. APPPS1:E4-SD
Figure 6I	APPPS1:E3-F-con: n=14;<br>APPPS1:E3-F-SD: n=14;<br>APPPS1:E3-M-con: n=13;<br>APPPS1:E3-M-SD: n=14;<br>APPPS1:E4-F-con: n=15;<br>APPPS1:E4-F-SD: n=14;<br>APPPS1:E4-M-con: n=15;<br>APPPS1:E4-M-SD: n=15;	Three-way ANOVA, apoE genotype $F(1,106) = 12.50$, $p < 0.001$; Sleep condition $F(1,106) = 12.35$, $p < 0.001$; apoE genotype X Sleep condition $F(1,106) = 4.861$, $p = 0.030$ in APPPS1:E3-con vs. APPPS1:E3-SD and APPPS1:E4-con vs. APPPS1:E4-SD
Figure 6J		Three-way ANOVA, apoE genotype $F(1,106) = 20.70$, $p < 0.001$; Sleep condition $F(1,106) = 12.53$, $p < 0.001$; apoE genotype X Sleep condition $F(1,106) = 5.645$, $p = 0.019$ in APPPS1:E3-con vs. APPPS1:E3-SD and APPPS1:E4-con vs. APPPS1:E4-SD
Figure 6K		Three-way ANOVA, apoE genotype $F(1,106) = 1.666$, $p = 0.200$; Sleep condition $F(1,106) = 5.726$, $p = 0.018$; apoE genotype X Sleep

		condition F(1,106) =2.136, p = 0.147 in APPPS1:E3-con vs. APPPS1:E3-SD and APPPS1:E4-con vs. APPPS1:E4-SD
Figure 6L		Three-way ANOVA, apoE genotype F(1,106) = 1.877, p = 0.174; Sleep condition F(1,106) =6.617, p = 0.011; apoE genotype X Sleep condition F(1,106) =5.178, p = 0.025 in APPPS1:E3-con vs. APPPS1:E3-SD and APPPS1:E4-con vs. APPPS1:E4-SD
Figure 6M		Three-way ANOVA, apoE genotype F(1,106) = 11.98, p < 0.001; Sleep condition F(1,106) =19.65, p < 0.001; apoE genotype X Sleep condition F(1,106) =3=0.2315, p = 0.631 in APPPS1:E3-con vs. APPPS1:E3-SD and APPPS1:E4-con vs. APPPS1:E4-SD
Figure 6N		Three-way ANOVA,apoE genotype F(1,106) = 14.60, p < 0.001; Sleep condition F(1,106) =24.62, p < 0.001; apoE genotype X Sleep condition F(1,106) =0.0002, p = 0.988 in APPPS1:E3-con vs. APPPS1:E3-SD and APPPS1:E4-con vs. APPPS1:E4-SD
Figure 7C	APPPS1:E3-F-con: n=15; APPPS1:E3-F-SD: n=14; APPPS1:E3-M-con: n=14; APPPS1:E3-M-SD: n=14; APPPS1:E4-F-con: n=15; APPPS1:E4-F-SD: n=14; APPPS1:E4-M-con: n=15; APPPS1:E4-M-SD: n=15;	Three-way ANOVA, apoE genotype F(1,108) = 26.82, p < 0.001; Sleep condition F(1,108) =24.74, p < 0.001; apoE genotype X Sleep condition F(1,108) =0.7273, p = 0.396 in APPPS1:E3-con vs. APPPS1:E3-SD and APPPS1:E4-con vs. APPPS1:E4-SD
Figure 7D		No significance, Three-way ANOVA, apoE genotype F(1,108) = 1.107, p = 0.295; Sleep condition F(1,108) =7.787, p = 0.006; apoE genotype X Sleep condition F(1,108) =3.293, p = 0.072 in APPPS1:E3-con vs. APPPS1:E3-SD and APPPS1:E4-con vs. APPPS1:E4-SD
Figure 8B-AQP4	APPPS1:E3-M-con: n=6; APPPS1:E3-M-SD: n=6; APPPS1:E4-M-con: n=6; APPPS1:E4-M-SD: n=6;	Two-way ANOVA, apoE genotype F(1,20) = 5.08, p = 0.0356; Sleep condition F(1,20) =1.709, p = 0.2060; apoE genotype X Sleep condition F(1,20) =10.71, p = 0.0038 in APPPS1:E3-con vs. APPPS1:E3-SD and APPPS1:E4-con vs. APPPS1:E4-SD
Figure 8B-TMEM119		Two-way ANOVA, apoE genotype F(1,20) = 0.9121, p = 0.3510; Sleep condition F(1,20) =14.97, p = 0.0010; apoE genotype X Sleep condition F(1,20) =0.3687, p = 0.5505 in APPPS1:E3-con vs. APPPS1:E3-SD and APPPS1:E4-con vs. APPPS1:E4-SD
Figure 8D	APPPS1:E3-M-con: n=4; APPPS1:E3-M-SD: n=4; APPPS1:E4-M-con: n=4; APPPS1:E4-M-SD: n=4;	Two-way ANOVA, apoE genotype F(1,12) = 2.980, p = 0.1099; Sleep condition F(1,12) =6.055, p = 0.0300; apoE genotype X Sleep condition F(1,20) =2.873, p = 0.1159 in APPPS1:E3-con vs. APPPS1:E3-SD and APPPS1:E4-con vs. APPPS1:E4-SD

Figure 8E		Two-way ANOVA, apoE genotype F(1,12) = 1.000, p = 0.3370; Sleep condition F(1,12) =5.023, p = 0.0447; APOE istofom X Sleep condition F(1,20) =2.098, p = 0.1731 in APPPS1:E3-con vs. APPPS1:E3-SD and APPPS1:E4-con vs. APPPS1:E4-SD
Figure 9C	APPPS1:E3: n=8 (4 males and 4 females); APPPS1:E3-AD Tau inj.: n=8 (4 males and 4 females); APPPS1:E4-AD Tau inj. : n=8 (4 males and 4 females); APPPS1:E4: n=9 (4 males and 5 females);	Three-way ANOVA, apoE genotype F(1,25) = 13.44, p = 0.0012; AD tau injection F(1,25) =94.84, p < 0.0001; apoE genotype X AD tau injection F(1,25) = 20.22, p = 0.0001 (14:00-17:00), apoE genotype F(1,25) = 4.2614, p = 0.0495; AD tau injection F(1,25) =27.96, p < 0.0001; apoE genotype X AD tau injection F(1,25) = 23.07, p < 0.0001 (18:00-21:00), apoE genotype F(1,25) = 38.31, p < 0.0001; AD tau injection F(1,25) =0.4444, p = 0.5111; apoE genotype X AD tau injection F(1,25) = 9.991, p = 0.0041 (00:00-03:00) in APPPS1:E3 vs. APPPS1:E3-AD Tau inj. and APPPS1:E4, and APPPS1:E4-AD
Figure 9D		Three-way ANOVA, apoE genotype F(1,25) = 9.694, p = 0.0046; AD tau injection F(1,25) =139.1, p < 0.0001; apoE genotype X AD tau injection F(1,25) = 59.59, p < 0.0001 (14:00-17:00), apoE genotype F(1,25) = 59.27, p < 0.0001; AD tau injection F(1,25) =213.6, p < 0.0001; Sex F(1,25) = 17.86, p = 0.0003; apoE genotype X AD tau injection F(1,25) = 58.24, p < 0.0001; apoE genotype X Sex F(1,25) = 7.566, p = 0.0109; apoE genotype X AD tau injection X Sex F(1,25) = 11.75, p= 0.0021 (18:00-21:00); apoE genotype F(1,25) = 102.4, p < 0.0001; AD tau injection F(1,25) =188.2, p < 0.001; Sex F(1,25) =8.712, p = 0.0068; apoE genotype X AD tau injection F(1,25) = 64.03, p < 0.0001; apoE genotype X Sex F(1,25) = 11.42, p = 0.0024; apoE genotype X AD tau injection X Sex F(1,25) = 10.73, p= 0.0031 (00:00-03:00) in APPPS1:E3 vs. APPPS1:E3-AD Tau inj. and APPPS1:E4, and APPPS1:E4-AD
Figure 9G	6M (control): n=9; 6M (AD tau inj.): n=6	No significance, Student’s t-test, t = 0.3569, p = 0.7298 (24 hour); t = 1.042, p = 0.3162 (Light); t = 1.479, p = 0.1629; (Dark) in 6M (control) vs. 6M (AD tau inj.)
Figure 9H		Student’s t-test, t = 1.395, p = 0.1864 (24 hour); t = 2.401, p = 0.0320 (Light); t = 1.938, p = 0.0747 (Dark) in 6M (control) vs. 6M (AD tau inj.)

Supplementary Table 1. Detailed statistical information for all figures.
